# On the Extraction and Analysis of Graphs From Resting-State fMRI to Support a Correct and Robust Diagnostic Tool for Alzheimer's Disease

**DOI:** 10.3389/fnins.2018.00528

**Published:** 2018-09-28

**Authors:** Claudia Bachmann, Heidi I. L. Jacobs, PierGianLuca Porta Mana, Kim Dillen, Nils Richter, Boris von Reutern, Julian Dronse, Oezguer A. Onur, Karl-Josef Langen, Gereon R. Fink, Juraj Kukolja, Abigail Morrison

**Affiliations:** ^1^Institute of Neuroscience and Medicine (INM-6), Institute for Advanced Simulation (IAS-6), JARA BRAIN Institute I, Jülich Research Centre, Jülich, Germany; ^2^Faculty of Health, Medicine and Life Science, School for Mental Health and Neuroscience, Alzheimer Centre Limburg, Maastricht University, Maastricht, Netherlands; ^3^Division of Nuclear Medicine and Molecular Imaging, Department of Radiology, Harvard Medical School, Massachusetts General Hospital, Boston, MA, United States; ^4^Department of Cognitive Neuroscience, Faculty of Psychology and Neuroscience, Maastricht University, Maastricht, Netherlands; ^5^Kavli Institute for Systems Neuroscience, Norwegian University of Science and Technology (NTNU), Trondheim, Norway; ^6^Cognitive Neuroscience, Institute of Neuroscience and Medicine (INM-3), Jülich Research Centre, Jülich, Germany; ^7^Department of Neurology, University Hospital of Cologne, Cologne, Germany; ^8^Cognitive Neuroscience, Institute of Neuroscience and Medicine (INM-4), Jülich Research Centre, Jülich, Germany; ^9^Department of Neurology, Helios University Hospital Wuppertal, Wuppertal, Germany; ^10^Faculty of Psychology, Institute of Cognitive Neuroscience, Ruhr-University Bochum, Bochum, Germany

**Keywords:** Alzheimer's disease, MCI, graph theory, resting-state fMRI, diagnosis, model by sufficiency, negative surprise

## Abstract

The diagnosis of Alzheimer's disease (AD), especially in the early stage, is still not very reliable and the development of new diagnosis tools is desirable. A diagnosis based on functional magnetic resonance imaging (fMRI) is a suitable candidate, since fMRI is non-invasive, readily available, and indirectly measures synaptic dysfunction, which can be observed even at the earliest stages of AD. However, the results of previous attempts to analyze graph properties of resting state fMRI data are contradictory, presumably caused by methodological differences in graph construction. This comprises two steps: clustering the voxels of the functional image to define the nodes of the graph, and calculating the graph's edge weights based on a functional connectivity measure of the average cluster activities. A variety of methods are available for each step, but the robustness of results to method choice, and the suitability of the methods to support a diagnostic tool, are largely unknown. To address this issue, we employ a range of commonly and rarely used clustering and edge definition methods and analyze their graph theoretic measures (graph weight, shortest path length, clustering coefficient, and weighted degree distribution and modularity) on a small data set of 26 healthy controls, 16 subjects with mild cognitive impairment (MCI) and 14 with Alzheimer's disease. We examine the results with respect to statistical significance of the mean difference in graph properties, the sensitivity of the results to model and parameter choices, and relative diagnostic power based on both a statistical model and support vector machines. We find that different combinations of graph construction techniques yield contradicting, but statistically significant, relations of graph properties between health conditions, explaining the discrepancy across previous studies, but casting doubt on such analyses as a method to gain insight into disease effects. The production of significant differences in mean graph properties turns out not to be a good predictor of future diagnostic capacity. Highest predictive power, expressed by largest negative surprise values, are achieved for both atlas-driven and data-driven clustering (Ward clustering), as long as graphs are small and clusters large, in combination with edge definitions based on correlations and mutual information transfer.

## 1. Introduction

The two major challenges in Alzheimer's disease (AD) research consist in firstly, finding an effective treatment that at least slows down the disease progress, and secondly, developing diagnostic tools that can not only detect the disease at the earliest stage, during which no symptoms related to cognitive deficits are apparent (Sperling et al., 2011), but also provide information into the progression of the disease. For the latter challenge it is particularly desirable that the tools can be deployed within the existing medical infrastructure (i.e., not requiring specialized machinery or lab procedures), such that it is feasible to scan a wide range of the elderly population. Diagnosis procedures currently in use include psychological tests, detection of abnormal concentrations of disease specific biomarkers (Amyloid-β, tau proteins) in cerebrospinal fluid and analysis of structural magnetic resonance images (MRI).

Although abnormalities of Amyloid-β concentrations are proposed to be the earliest disease indicator, they are not very reliable in disease prognosis. Moreover, the changes in Amyloid-β concentrations show the strongest increase in the preclinical phase, and are thus uninformative with respect to the further progression of the disease. Tau pathology, which probably spreads along functional networks (Hoenig et al., 2018) better predicts cognitive deficits and progression of the disease (Nelson et al., 2012). However, the two methods measuring Amyloid-β and tau concentrations, lumbar puncture and PET are invasive (Schroeter et al., 2009; Sperling et al., 2011).

Possibly, synaptic dysfunction, another disease marker, corresponds to the onset of AD even before Amyloid-β pathology starts. Additionally, as it gradually worsens throughout the course of the disease, it could serve as diagnostic marker for all stages of AD. Dysfunction of synapses can be indirectly measured via invasive FDG-PET and non-invasive functional MRI, which might directly be combined with structural MRI scans (Schroeter et al., 2009; Sperling et al., 2011). However, a diagnostic framework based on functional MRI has yet to be established.

Although many fMRI studies have investigated changes of functional activity in AD (for a review see Dennis and Thompson, 2014), there is no consensus about which information should be used. Such studies typically examine disrupted cortical connectivity, either locally, considering single brain areas (e.g., Dillen et al., 2017) and their embedding in the network, or globally, analyzing the entire constructed brain graph and the statistics of its graph properties (Gits, 2016).

We argue that in order to develop a robust diagnosis tool applicable to all disease stages, it is preferable to consider global graph properties for the following reasons. First, global graph properties seem to be more robust across sessions; consequently, changes in these properties over time are more likely to reflect disease progression than statistical fluctuations (Telesford et al., 2010; Wang et al., 2014). Second, not all disease progressions follow a stereotypical pattern. Whereas structural evidence of AD is typically found predominantly in entorhinal cortex and hippocampus, in atypical cases atrophy occurs primarily in other areas, such as posterior cortex (Johnson et al., 2012). These atypical cases might be better captured by global properties, since they make use of the entire information provided by the brain. Furthermore, analyzing the statistics of graph properties rather than comparing the properties of single nodes allows the use of data-driven brain clustering, which results in different numbers and locations of brain clusters for each individual.

However, it is challenging to investigate the informativeness of global graph properties due to the innumerable methods of graph construction, comprising both the clustering of the voxels to define the graph's nodes, and the definition of functional connectivity to define its edges. Across the range of previous studies investigating graph properties in AD, a wide variety of methodological approaches for graph construction and properties assessment have been applied and are probably a major source of contradictory observations, such as the comparative length of the shortest path in AD subjects with respect to control being reported in two recent studies as both shorter (Zhao et al., 2012) and longer (Sanz-Arigita et al., 2010).

It is a further challenge to identify an appropriate evaluation method that not only enables us to compare the different graph construction methods, but also permits the results to be combined with other information indicating the probability of a particular health condition. This means that pure classifiers, although they achieve high discrimination performance (Khazaee et al., 2015, 2017) do not meet these requirements because they return a group membership (“AD,” “MCI,” or “control”) and not a probability that can be combined with the results of other diagnostic tests (e.g., derived from Amyloid-β concentration measures) or individual patient risk factors (Porta Mana et al., 2018).

In this article, we address these issues by presenting a methodology for determining which combination of techniques to extract and analyze graphs from resting state fMRI data provides the best basis for a diagnosis tool, assuming a given initial data set. Here, we apply our methodology to a small data set consisting of 26 control (C) elderly patients without any indication of any form of dementia or other cognitive problems, 16 mild cognitive impaired (MCI) subjects and 14 patients suffering from Alzheimer disease (AD) (Dillen et al., 2017). We evaluate the combinations of graph construction and analysis methods using a statistical model that partly compensates for the small data set and also yields probabilities rather than classifications, thus permitting the results to be combined with other probabilities, as discussed above. In addition, we evaluate the graph construction techniques with respect to robustness of results to method configuration parameters and similarity of results across different techniques.

Note that our aim here is not to demonstrate superior classification (for which our data set is in any case too small) or to propose a particular combination of techniques as optimal (as this may vary between settings), but primarily to provide a principled way for determining an appropriate combination of techniques for a given data set, and secondarily to highlight the sensitivity of graph theoretical analysis to the details of graph construction.

**Figure 1:**
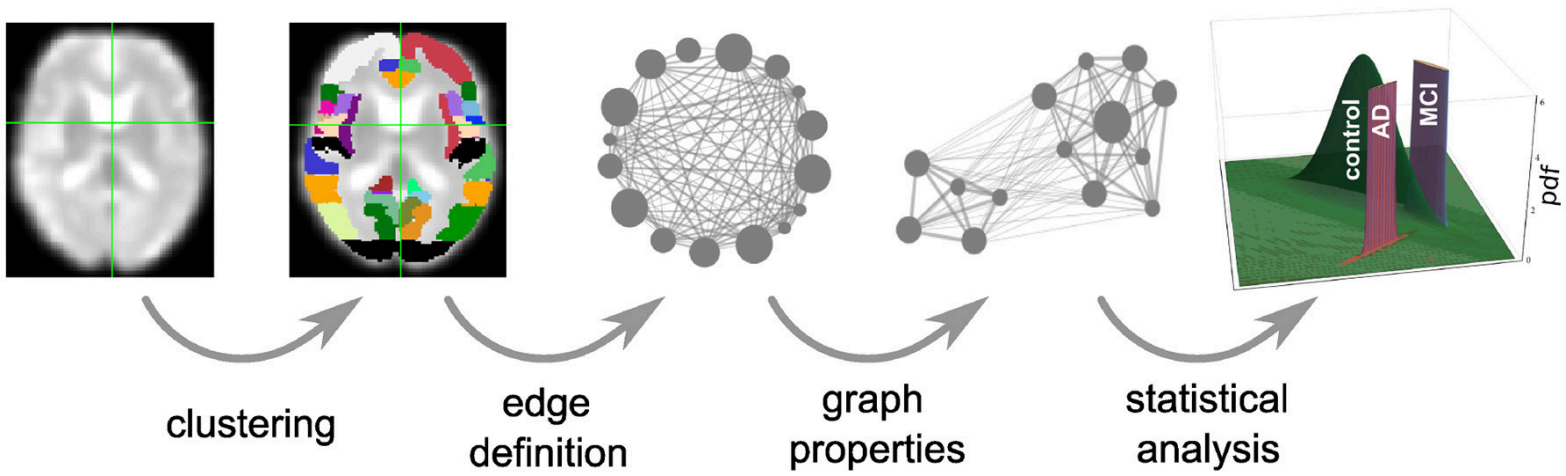
Overview of intermediate steps for graph construction, properties derivation and statistical analysis. Each picture illustrates the result of a processing step starting from the preprocessed functional image (far left), which is clustered into regions, used as the nodes of the graph (second image). The averaged fMRI activity of each region is then used to calculate the edges of the graph (third image) and based on the calculated graph properties (fourth image) of all graphs, the statistical analysis estimates the probability density functions (pdf) of the three health conditions (last image) that are necessary for the evaluation of diagnostic performance based on the negative surprise measure. For the first three steps of the pipeline we investigate a range of different methods, see sections 2.1, 5.3, 5.4, and 5.5 for details.

To understand how different methods for constructing graphs affect the resultant graph properties, and thus the ability to distinguish between patient groups, we evaluate a range of standard and non-standard methods to construct the graphs. The first step in graph construction consists in clustering adjacent voxels, such that the activity of the resulting region can be expressed by the average of time varying signal of the selected voxels (see Figure 1). The decision as to which voxels form a cluster is often based on atlases established for a standard brain with predefined brain regions. In order to map this standard atlas to the functional image or vice versa, registration algorithms are used. Problematic in this step, especially for subjects potentially suffering from neurodegenerative diseases, is the inhomogeneous shrinkage of the brain, which hampers a correct registration (Liu et al., 2017). In addition, individual brain regions derived from standard brain templates are likely to execute several cognitive processes in parallel, such that averaging the activity across the voxels of these functional inhomogeneous regions is not justified (Marrelec and Fransson, 2011). We therefore also include activity driven algorithms, namely region growing and selection (Lu et al., 2003) and Ward clustering, into our evaluation.

In the second step in graph construction, functional connectivity values are calculated based on the averaged signal of the regions. In most studies this is carried out based on the Pearson correlation coefficient, restricting the functional connectivity to non-directional connections. Here we cover a broader range of possible measures in the time domain: linear, non-linear model-free and model-based (Wang et al., 2014) that, depending on their exact realization, result in directed or undirected graphs.

We then calculate a variety of graph measures on the single nodes (weighted degree, cluster coefficient, closeness centrality), edges (weights, shortest path) and the entire graph (modularity). As several of these measures are only well-defined for binary graphs, many studies binarize the weighted graphs obtained from the previous steps into binary graphs, by setting weights above an arbitrary threshold *w*min to 1, and those below it to 0 (e.g., Supekar et al., 2008). The drawback here is that there is no validation for an optimal threshold, and information that might be relevant in AD may be lost. To investigate this problem, we analyze the dependence of graph theoretic measures on *w*min, setting the weights below it to 0 but leaving the values above unchanged.

To assess the suitability of combinations of graph construction and analysis methods to inform a diagnosis tool, we set up a statistical analysis based on a training data set of known health conditions (healthy controls, mild cognitive impairment, and Alzheimer's disease), see section 5.6. The diagnostic usefulness of the analysis pipeline is then defined as the performance of the model against a labeled test data set. A model with good performance can ultimately be employed in a clinical setting, to assess the probability that a patient has one of the three health conditions. For a more complete discussion of the development and use of the statistical model, see Porta Mana et al. (2018).

In this study we use a statistical model constructed from the following working hypothesis: the empirical means and correlations of graph data from previous patients with a given health condition are sufficient to predict the graph data of a new patient with that same health condition. This is a partially exchangeable model by sufficiency, and the resulting likelihood is a multivariate t distribution (Porta Mana et al., 2018), described in section 5.6. To assess which graph constructions have the greatest predictive power, we calculate their log-probabilities or *negative surprises* (Bartlett, 1952; Good, 1956, 1957a,b, 1983). To validate this approach, we also compare the results of the negative surprise with the classification performance achieved by a support vector machine (SVM).

Our results show that clustering resulting in small graphs with large clusters (Ward and atlas-based clustering) achieve highest negative surprises (and best SVM classification performance). Similarly, amongst the edge definition techniques, model-free methods (linear and non-linear correlations, mutually information transfer) obtain the highest negative surprise values. Conversely, calculating the graph's edge weights according to transfer entropy (model based) achieves limited diagnostic power but the ordering of the individuals based on their average graph properties is very robust toward the applied clustering method and choice of algorithm specific parameters. We further demonstrate that significant differences in the means of graph properties are very sensitive to method choice and to parameterization choices for a given method. Therefore such results, if taken at face value and not validated by alternate methods, may well be artifactual and not provide insight into the effects of a disease. Interestingly, the presence of significant differences in mean values of graph properties is not a reliable predictor of later diagnostic performance. In particular, atlas clustering results in only few significant differences but reaches the highest values for negative surprises and the best classification scores for the SVM. Finally, we show that the effect of setting a threshold on the graphs edge weights has only marginal effect on the negative surprise as long as threshold values are small.

## 2. Results

### 2.1. Graph construction

#### 2.1.1. Vertex definition by means of clustering

A universal property of the clustering algorithms examined here is the existence of a control parameter that regulates how the clusters are formed, and thus preserves a certain feature (or features) of the clusters. In atlas-based clustering, the preserved features are the number of clusters and the number of voxels per cluster. In Ward clustering, the number of resulting clusters is fixed, which we violate to a small extent by deleting very small clusters. In region growing and selection (RGS), the homogeneity of each cluster is preserved. The freedom that each of the algorithms leaves to the non-regulated features can either be considered as a drawback of the algorithm, because it makes graphs less easily comparable, or as an additional feature that might even improve the diagnosis performance.

**Figure 2:**
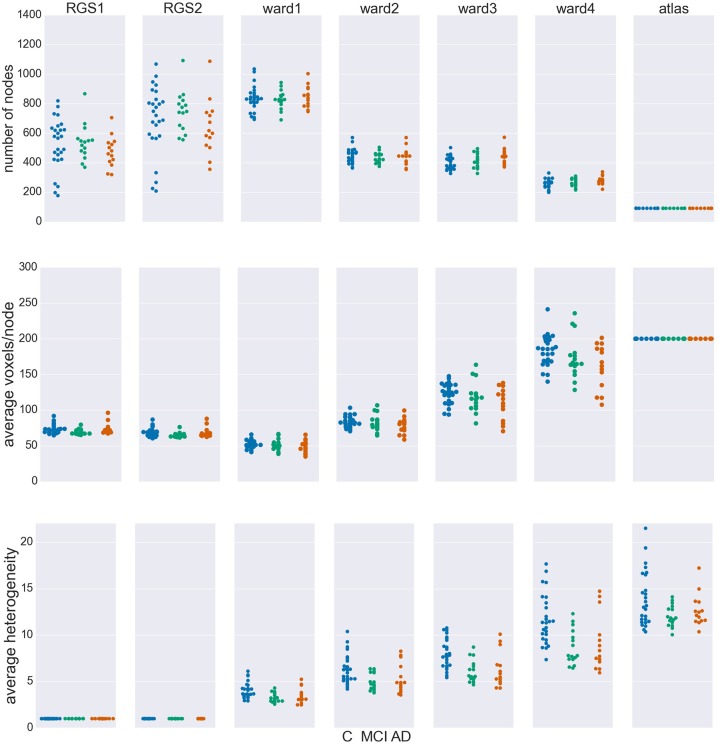
Node properties across different clustering algorithms. For each of the seven clustering methods detailed in section 5.3 and Table 3, and each subject categorized in the health conditions: control (C, blue dots), mild cognitive impairment (MCI, green dots), and Alzheimer's disease (AD, orange dots) we calculate the total number of nodes/clusters generated **(Upper Panel)**, the average number of voxels per node **(Middle Panel)**, and the average cluster heterogeneity **(Lower Panel)**.

Figure 2 shows the number of nodes/clusters, the average number of voxels per node and the average heterogeneity of the nodes for two configurations of the RGS algorithm, four configurations of the Ward algorithm, and the atlas algorithm (see section 5.3 and Table 3). Most strikingly, the node properties vary far more with respect to the clustering method chosen than with respect to the health condition.

By construction, the number of nodes for atlas clustering are the same for all individuals, and are the smallest over all the clustering methods (top panel). In Ward clustering the number of clusters is a parameter of the algorithm; it is not constant in Figure 2 because we additionally include a parameter enforcing a minimum cluster size. Thus, the number of nodes for Ward clustering decreases as the minimum number of voxels per cluster *p* increases from 10 for “ward1” to 25 in “ward4.” In RGS clustering we do not have such restrictions and the number of clusters is defined by the voxel dynamics. A consequence of this is that the number of clusters per graph are more widely spread.

The average number of voxels per cluster, shown in the middle panel of Figure 2, is unsurprisingly negatively correlated with the number of clusters. For purposes of comparison, the number of voxels for atlas clustering was first calculated for the standard space and then downscaled in proportion to the relation of the total number of voxels present in functional space to those in standard space. An inverse correlation can also be seen in the width of the distributions between the top two panels, for the non-atlas methods. In the case of RGS clustering, this can be explained by the fixation of the heterogeneity to one (see bottom panel of Figure 2), leading to quite homogeneous numbers of voxels per cluster, but to a wide range of the number of nodes, namely from 200 to 1200. Since this range is so large, it could be argued that graph properties that depend on this number would not be comparable in a meaningful fashion. In order to take care of such dependencies, we include the number of nodes in our statistical analysis (section 5.6). For Ward clustering we can observe that the numbers of nodes is inversely correlated not only with the average number of nodes and its variability, but also with the average heterogeneity and its variability. We observe the highest degree of heterogeneity for atlas clustering, presumably due to the high number of voxels per cluster.

Comparing node properties between the classes of clustering methods, atlas and ward4 clustering seem to be quite similar, which suggests they might result in similar graph properties and diagnosis performance. In particular, we note that these methods reveal a much smaller heterogeneity for the MCI group than for the control and AD groups.

#### 2.1.2. Edge definition by means of functional connectivity

The edges of the graphs are constructed in four different ways, described in detail in section 5.4. Linear correlations (*corr*) are based on the Pearson correlation coefficient; non-linear correlations (*H*_2_) result from a non-linear fit of piecewise linear correlations; mutual information transfer (*MIT*) measures the amount of shared information between two time varying signals and transfer entropy (*TE*) describes in how far the future uncertainty is reduced by the preceding activity of the considered pair of nodes. As with the clustering algorithms described in the previous section, we defined differently parameterized variants of these four classes of technique (e.g., generating directed *D* or undirected *U* graphs) which are listed in Table 4.

For each combination of vertex (RGS, Ward or atlas) and edge definition technique (*corr*, *H*_2_, *MIT*, *TE*), we averaged over the weights generated in each health condition for each variant of both techniques. For example, for the combination of region growing and transfer entropy (RGS *TE*) we averaged over all combinations of clustering implementation (RGS1 and RGS2) and edge detection (*BTEU*1, *BTEU*2, *BTED*1, *BTED*2). The results are shown in Table 1 and exhibit a high variability in the mean connection weights. For instance, the combination RGS *TE* yields a maximal mean weight of 0.158 for controls, which is three times lower than the maximum mean weight of 0.493 obtained by the RGS *H*_2_ combination. In particular, RGS clustering yields higher values compared with Ward and atlas clustering for model-free edge definitions (*corr*, *H*_2_, *MIT*). The smallest values are obtained for *TE*. As a consequence, even small thresholds e.g., *w*min = 0.3 already cause *TE* graphs to disintegrate. Accordingly, not all graph properties can be calculated and used for statistical analysis, as shown in section 2.3.

**Table 1:** Mean and standard deviation of edge weight across different edge definitions.

**Combination**	**ŵ_*C*_**	**ŵ_*MCI*_**	**ŵ_*AD*_**
Ward *corr*	0.328 ± 0.021	0.337 ± 0.04	0.315 ± 0.023
RGS *corr*	0.405 ± 0.076	0.363 ± 0.049	0.397 ± 0.113
atlas *corr*	0.319 ± 0.02	0.334 ± 0.054	0.307 ± 0.022
Ward *H*_2_	0.443 ± 0.18	0.398 ± 0.081	0.414 ± 0.057
RGS *H*_2_	0.452 ± 0.1	0.471 ± 0.126	0.493 ± 0.179
atlas *H*_2_	0.36 ± 0.057	0.352 ± 0.039	0.355 ± 0.042
Ward *MIT*	0.201 ± 0.004	0.2 ± 0.007	0.197 ± 0.004
RGS *MIT*	0.221 ± 0.026	0.204 ± 0.011	0.218 ± 0.037
atlas *MIT*	0.196 ± 0.003	0.197 ± 0.008	0.193 ± 0.003
Ward *TE*	0.163 ± 0.013	0.158 ± 0.015	0.156 ± 0.018
RGS *TE*	0.158 ± 0.026	0.149 ± 0.02	0.152 ± 0.042
atlas *TE*	0.163 ± 0.016	0.17 ± 0.011	0.165 ± 0.011

*Means and standard deviations are taken across the average edge weight of every individual graph in a health condition. Highest mean edge weights for each combination across the three health conditions are highlighted in gray*.

**Figure 3:**
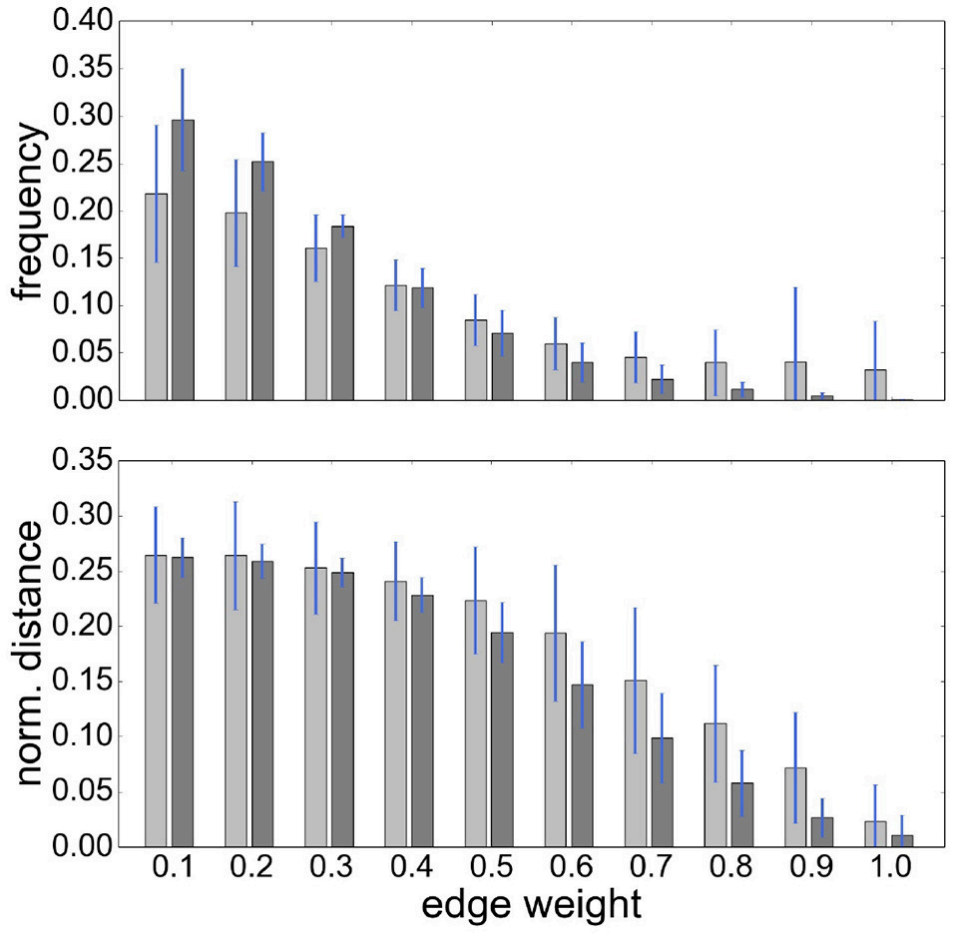
RGS clustering yields stronger long-range connections then Ward clustering. Frequency **(Upper Panel)** and connection distance normalized to maximum graph distance **(Lower Panel)** across a range of graph edge weights calculated based on *BcorrU*1 for RGS1 (light gray bars) and ward2 (dark gray) clustering. Mean values and standard deviation (blue vertical lines) are calculated across single histogram values of all subjects independent of health condition.

It is also notable that there is no systematic relationship between the three health conditions—for RGS *corr*, the control graphs have the highest mean weight, for RGS *H*_2_, the AD graphs; and for atlas corr, the MCI graphs. These results demonstrate that conclusions drawn on health conditions based on weight statistics should be treated with suspicion, as the outcome can be strongly influenced by the method of calculation. A possible explanation for the higher weights generated by RGS clustering is that it produces a greater number of shorter distances compared with the other clustering techniques. However, although Figure 3 does indeed confirm that edge weights become smaller with cluster distance, it does not reveal a bias to shorter weights for RGS. In fact, the converse is true: RGS clustering yields stronger long-range connections for similar graph sizes [average number of graph nodes: 379.69 ± 147.99 (RGS), 311.43 ± 33.59 (Ward); average edge weights for distances longer than 0.8: 0.25 (RGS), 0.18 (Ward)]. Therefore we conclude that connecting homogeneous clusters allows stronger long-range connections to be extracted. However, the statistics of the RGS connections has a much larger variance then the ones derived from Ward clustering. This is only partly due to the variance in the number of nodes, since even if we choose three healthy subjects with similar graph size (RGS: 297±2.16, Ward: 297.33±6.6), we still get a higher standard derivation for RGS clustering in the weight distribution (σ_RGS_/σ_ward_ = 1.6).

In the following we will treat the distribution of edge weights as a graph property since it contains information about graph structure.

### 2.2. Graph properties

**Figure 4:**
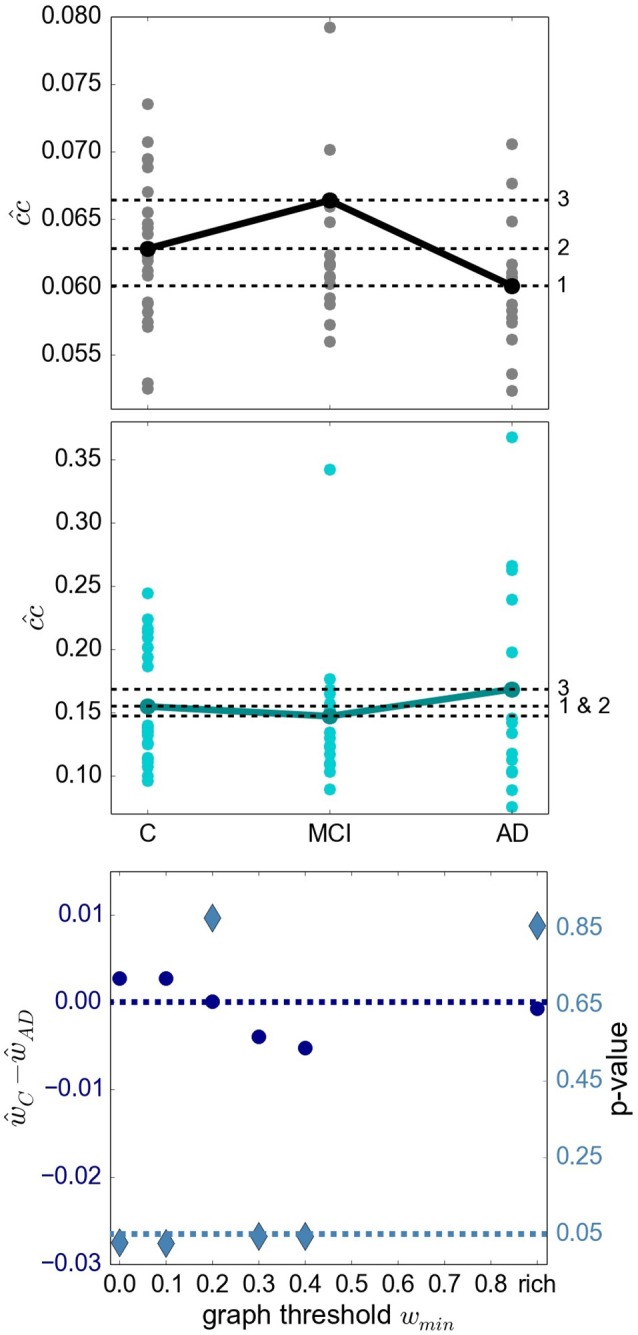
Relationship of sub-graph properties across heath conditions is dependent on graph size. **(Upper Panel)** Average closeness centrality $\hat{cc}$ across graph nodes for complete graphs constructed with atlas *BMITU1* for the different health conditions C (left), MCI (middle) and AD (right). Each dot corresponds to the graph of an individual (connected dots indicate the mean values). **(Middle Panel)** As in top panel, but on the basis of the rich club graphs. **(Lower Panel)** Difference of the averaged ward1 *BMTID*2 graph weights of the control group ŵ_*C*_ and the AD group ŵ_*AD*_ (left vertical axis, blue discs) and significance of this difference (right vertical axis, turquoise diamonds) as functions of the graph thresholding value *w*min. All ŵ are positive and are only calculated as long as graphs are connected (which is the case for *w*min < 0.5). Average is taken across the weights of individual graphs. The dashed dark blue line indicates ŵ_C_−ŵ_AD_ = 0; the dashed turquoise line indicates a significance level of 0.05.

A recent survey by Gits (2016) of studies investigating graph properties in AD reveals no clear and systematic differences between heath conditions. For example, the mean clustering coefficient was found to be both significantly smaller (Supekar et al., 2008) and larger (Zhao et al., 2012) in AD compared to the aged-matched control group. We consider it likely that differences in methodology account for many of the contradictions. However, the stage of AD reached by the examined subject group may also play an important role. To investigate this aspect more closely, we examine the finding by Kim et al. (2015) that local efficiency, which corresponds to our definition of closeness centrality divided by the number of nodes in the network minus one, is increased for MCI, decreased for initial stages of AD and increased for severe AD stages with respect to the control group. The results of applying similar methods (atlas-based clustering combined with *BMITU*) are shown in Figure 4. The top panel shows the relationship between the health conditions when closeness centrality is calculated on the full, non-thresholded graph, which reproduces the findings of Kim et al. (2015), at least for initial stages of AD. However, if the measure is calculated on the graphs' rich club, i.e., the sub-graphs consisting of the nodes in the top 10% for degree, a different picture emerges, as shown in the middle panel of Figure 4. Here, AD has an increased closeness centrality with respect to both the control and mild cognitive impairment groups, which is in line with advanced AD stages in Kim et al. (2015).

More evidence that the outcome of a graph theoretical analysis can be highly sensitive toward the exact methodological implementation is given by considering the difference between the mean weights in the control and the AD conditions, and its significance (section 5.7.1), in dependence on the thresholding weight used to convert weighted graphs into simple graphs. This is illustrated in the bottom panel of Figure 4. Here, depending on where we set the threshold for considering an edge to be relevant, results having a significance level of *p* < 0.05 can be observed for both ŵ_C_>ŵ_AD_ (*w*min∈{0.0, 0.1}) and ŵ_C_ < ŵ_AD_ (*w*min∈{0.3, 0.4}).

**Figure 5:**
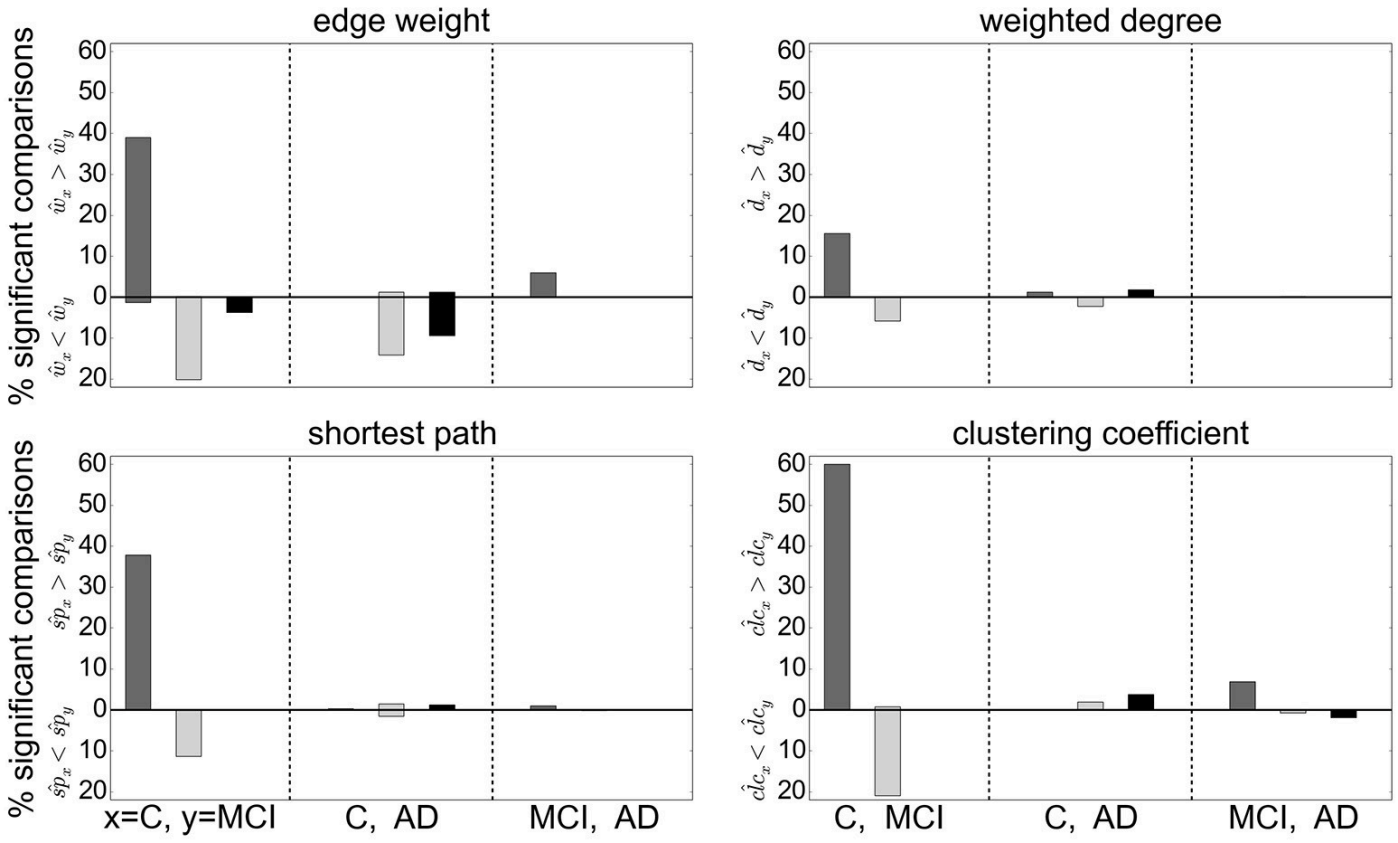
Significant relationships in graph metrics between health conditions dependent on clustering methods. Percentage of significant differences for each clustering method RGS (dark gray), Ward (light gray), and atlas (black) for different averaged graph properties: edge weight **(Upper Left)**, weighted degree **(Upper Right)**, shortest path **(Lower Left)** and clustering coefficient **(Lower Right)**. Fraction of significant differences are calculated for each health condition over all graphs constructed with the corresponding clustering including all variants in parameters, edge definition techniques, thresholds and rich club sub-graphs. The abscissa labels show which pairs of health conditions are compared (C-MCI, C-AD, MCI-AD) and the ordinate labels the direction of the significant differences (“<,” “>”). Significance is calculated as in the lower panel of Figure 4.

Extending this analysis, we find that contradictory significant results can be obtained for a variety of graph metrics across (and sometimes within) clustering methods. Figure 5 shows the percentage of significant results obtained for health condition relationships in average edge weight, weighted degree, shortest path and clustering coefficient. Most strikingly, for most examined relationships, if significant differences are found at all, they are found in both directions, e.g., both for ${\hat{d}}_{\text{C}}>{\hat{d}}_{\text{MCI}}$ and for ${\hat{d}}_{\text{C}}<{\hat{d}}_{\text{MCI}}$ (weighted degree). Often a clustering algorithm favors a particular comparison direction, e.g., for the clustering coefficient, RGS clustering yields ${\hat{clc}}_{MCI}>{\hat{clc}}_{AD}$ whereas Ward and atlas clustering yields ${\hat{clc}}_{MCI}<{\hat{clc}}_{AD}$. However, we also find cases where significant differences are found in both directions with approximately equal frequency, such as ${\hat{sp}}_{C}>{\hat{sp}}_{AD}$ and ${\hat{sp}}_{C}<{\hat{sp}}_{AD}$ for Ward clustering. In addition, we find some clustering algorithms show a systematic behavior across metrics, e.g., for RGS ${\hat{x}}_{C}>{\hat{x}}_{MCI}$ with *x*∈{*w, d, sp, clc*}.

The largest number of significant differences is found for the comparison of controls with MCI, followed by the comparison of controls with AD. Only few significant differences of the means are found for AD and MCI. This relation among the groups is in line with the observed differences in heterogeneity observed for Ward and atlas clustering, for which MCI showed much lower heterogeneity and AD slightly lower values compared to controls (bottom panel of Figure 2).

**Figure 6:**
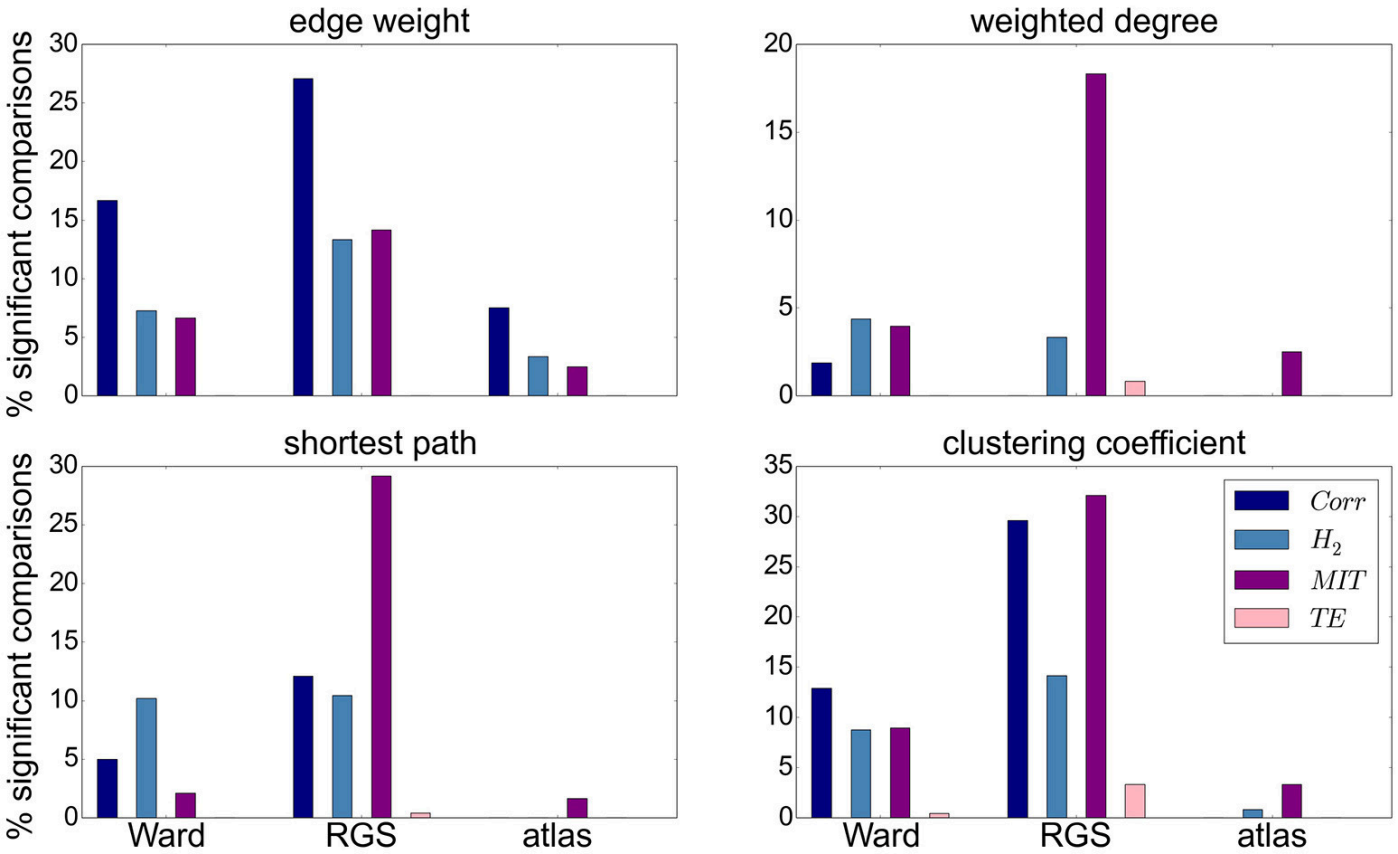
Significant relationships in graph metrics between heath conditions dependent on edge definition methods. Percentage of significant differences for each clustering technique [Ward (left), RGS (middle), atlas (right)] for each class of edge definition method clustering method [*corr* (dark blue), *H*_2_ (light blue), *MIT* (purple), *TE* (pink)] for averaged graph properties: edge weight **(Upper Left)**, weighted degree **(Upper Right)**, shortest path **(Lower Left)** and clustering coefficient **(Lower Right)**. Fraction of significant differences are calculated for each health condition over all graphs constructed with the corresponding clustering and edge techniques including all variants in parameters, thresholds and rich-club sub-graphs. Significance is calculated as in the lower panel of Figure 4.

Focusing on the clustering methods that bring about the most significant differences comparing the entire graph properties distributions results, we find the highest fraction for RGS, followed by Ward clustering. Atlas-based clustering yields only a few significant results. Figure 6 shows the breakdown of the proportion of significant results for each clustering method on the edge definition technique (shown in collated form in Figure 4). Notably, transfer entropy (*TE*) only rarely produces significant differences. All other edge definition methods show a similar fraction of significant comparisons. The highest number of significant comparisons across the different graph properties is generated by RGS clustering combined with *MIT*.

To what extent a greater proportion of significant relationships is likely to make this graph construction method a good basis for a diagnostic tool depends on two aspects. First, the significance test is performed only on mean values, but ideally the overall distributions should overlap as little as possible. Second, the correlation between graph properties should be small in order to avoid redundant information.

In this section we considered only the first moments (means) of the graph properties taken from an individual brain. However, as explained in section 5.5, we use the first four moments of the individual distributions for our statistical analysis. Since the *p*-value of the other moments is not calculated, its influence on the statistical analysis cannot be considered.

**Figure 7:**
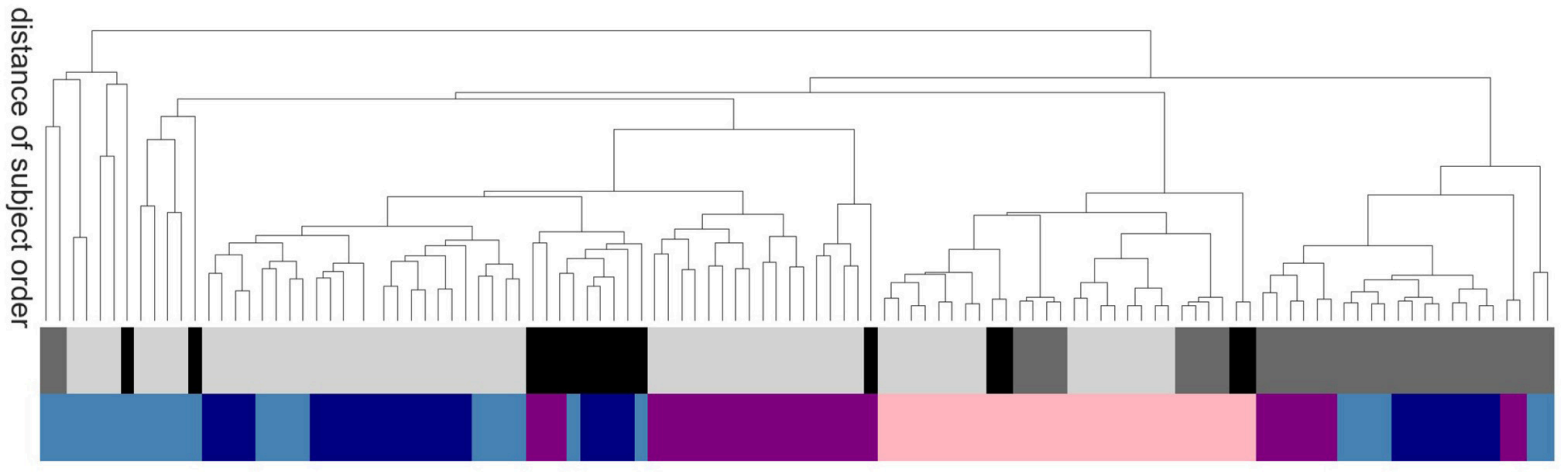
Sensitivity of subject order to clustering and edge detection techniques. The dendrogram shows the distance of subject order, calculated by ordering all subjects according to their average graph edge weights and calculating the Euclidean distance between the resulting rank arrays. For better legibility, instead of naming the dendritic leaves, of which every leaf corresponds to a particular combination of clustering and edge definition techniques, e.g., ward2 *BTED*2, the top row of colors code for the class of clustering method: Ward (light gray), RGS (dark gray) and atlas (black); and the bottom row codes for the class of edge definition method: *corr* (dark blue), *H*_2_ (light blue), *MIT* (purple) and *TE* (pink).

In order to evaluate the methods based on robustness due to methodical variation, we investigate how the order of subjects (all subjects independent of their health conditions are ordered according to their average value of a certain graph property) is affected by the exact realization of the graph construction methods. Graphs constructed by methods based on similar underlying features of the data will tend to show a systematic ordering of subjects, regardless of the absolute values of the calculated graph metrics. Figure 7 shows the commonalities and differences, which are illustrated with a dendrogram (see section 5.7.2) calculated on the Euclidean distance between the resulting ordered arrays of average graph weights. The continuous pink area show that graphs constructed using transfer entropy are most robust to the choice of clustering technique. Moreover, linear and non-linear correlations (dark and light blue) occupy contiguous blocks and so are most similar to each other. The leaves denoting atlas clustering (black) are rather spread out, indicating a high sensitivity of this method to the choice of edge definition.

In this section we have shown that the relationship of graph properties between health conditions strongly depends on the methods used for graph construction. For our data we find more significant mean differences for control-AD and control-MCI then for MCI-AD. With respect to clustering and edge definition methods, the largest number of significant differences are found for RGS and Ward clustering, and for model-free edge definitions. These results show that conclusions on how graph properties change due to AD have to be drawn carefully, and ideally validated by other methods, as they can be highly sensitive to the methods used for graph construction.

### 2.3. Evaluation of graph construction methods based on negative surprise

Having examined the consequences of particular choices for clustering and edge definition techniques in the previous sections, we now evaluate their combinations by considering their ability to help a clinician to discriminate among patient groups. This discrimination is achieved by using the graph data within a statistical model, which specifies the likelihood of the graph data. The model is described in section 5.6; the likelihood is a distribution which depends on a set of parameters. In general, the kind of graph data—i.e., their construction method—and the statistical model with its parameters are interdependent: they cannot be freely varied separately. Therefore our evaluations of the predictive power of the various graph construction methods have to be understood with a caveat: they depend on our specific choice of statistical model.

To quantify the discriminating power for each graph construction combination, we use a metric based on the final probabilities for the correct health conditions known as the log-probability, or *negative surprise* (Bartlett, 1952; Good, 1956, 1957a,b, 1983): a sure event, i.e., with unit probability, has surprise equal to zero; whereas an impossible event, i.e., with zero probability, has surprise equal to infinity, reflecting the fact that its occurrence would be contrary to all our expectations. A high surprise (in absolute value) therefore signals a low predictive power of the data we are using. The expectation or average of the surprises is the Shannon entropy (Shannon, 1948; Bartlett, 1952; McCarthy, 1956; Bernardo, 1979; Jaynes, 2003: section 11.3).

Another possibility, of a more decision-theoretical character, is to consider a metric based on the average utilities obtained with each particular graph-construction method. Given several possible courses of action (e.g., treat or dismiss) and their utilities or costs with respect to each health condition (e.g., treating an Alzheimer patient, dismissing a healthy patient, dismissing an Alzheimer patient, or treating a healthy one), the clinician should choose the action that maximizes the expected utility, the expectation being calculated from the final probabilities for the possible health conditions (Sox et al., 2013). This kind of metric therefore requires not only the final probabilities—which depend on the graph-construction method—but also a table of utilities.

Numerical tests show that the two kinds of metric yield similar results, at least for utility tables close to the identity (treating an ill patient and dismissing a healthy one have unit utility; the remaining combinations have zero utility). We therefore choose a metric based on the negative surprise, which is simpler and more intuitive than a utility metric.

In order to have an approximate idea of the relative predictive powers of the graph-construction methods we would like to use a statistical method that can be kept the same, as much as possible, across different methods. For this reason we choose a model based on the working hypothesis of sufficiency of mean and correlations of past data, as explained in the Introduction. This model ignores any restricted range of variability of graph quantities (e.g., positive or bounded). As explained in Porta Mana et al. (2018), this choice is non-standard but does not entail contradictions. The model has some free parameters; their values reflect the fact that the units of measure for the graph quantities make the latter of order unity. This choice of a generic, common statistical model allows us to sidestep the demanding problem of tailoring it for the different graph quantities from our 850 graph-construction methods.

**Figure 8:**
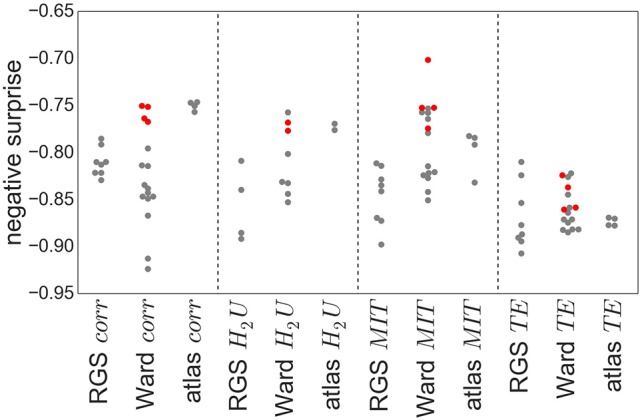
Negative surprise of the different graph construction methods. Each dot represents a specific node clustering (e.g., RGS1) and edge definition (e.g., *BcorrU*1). Dots are grouped together according to their main class (e.g., RGS *corr*). Red dots highlight the results of ward4 clustering. Negative surprise expected by chance is −1.1.

Figure 8 shows the obtained negative surprises for all combinations of graph construction methods except *H*_2_*D*, which is left out due to an inadequacy of the statistical model, resulting in unrealistic values between −1.26 and −0.66 with a mean and standard deviation of −0.94±0.19.

The differences in negative surprise between the different graph construction method are in general small. The best results are obtained for ward4 clustering combined with mutual information (*MIT*) based edge definition. Across edge definition methods, linear correlation (*corr*) and mutual information give the best results and transfer entropy (*TE*) the worst. The rather poor performance of *TE* edge definition is in line with the small number of significant differences found for this method (compare Figure 6). Comparing the different clustering methods, atlas and ward4 clustering give the best results, as long as the edge definition is not *TE*. These two clustering methods have in common a very small number of graph nodes and (correspondingly) the highest number of voxels per cluster (compare Figure 2).

As explained above, the comparison of graph-construction methods can be affected by the statistical model and its parameters, especially for small datasets. As a complementary analysis we compare the negative surprises with the classification performances of a support vector machine (SVM, section 5.7.1) based on the same graph constructions. In a clinical setting, a misclassification between control and AD has more severe consequences than between MCI and AD. To avoid introducing an asymmetric misclassification penalty, we perform the classification between pairs of classes only (control-AD, C-MCI, MCI-AD).

**Figure 9:**
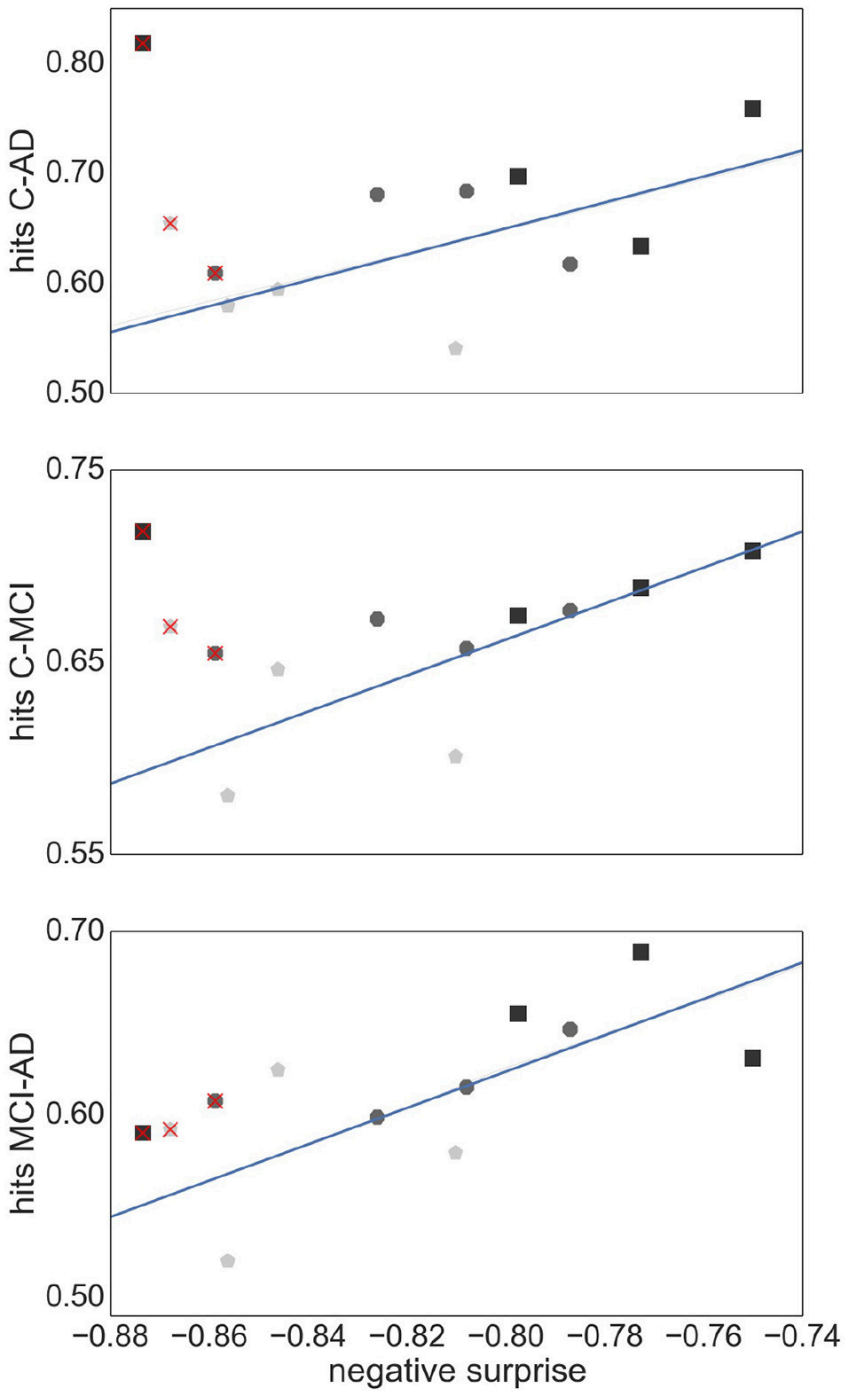
Relationship between SVM classification performance and negative surprise. The average SVM performance achieved by each combination of clustering method and edge definition with respect to each pair of health conditions: control-AD **(Upper Panel)**, control -MCI **(Middle Panel)**, and MCI-AD **(Lower Panel)**, is plotted against the negative surprise calculated for all health conditions. Each marker corresponds to the averaged performance across the parameter space of a specific clustering method [atlas (black squares), Ward (dark gray octagons), RGS (light gray pentagons)] and a specific edge defintion (*corr*, *H*_2_, *MIT*, *TE*). The regression line is calculated for all points but *TE* (superimposed red crosses). Pearson correlation coefficients *r* of the datasets are *r* = 0.59 **(Upper Panel)**, *r* = 0.77 **(Middle Panel)**, *r* = 0.69 **(Lower Panel)**.

Figure 9 shows the relationship between the SVM performance (measured as proportion of correct classifications) against the negative surprise. As long as *TE* edge definition is excluded, the two performance measures are positively correlated. In particular RGS clustering achieves low performance in both negative surprise and SVM classification. Furthermore, atlas clustering achieves a high classification performance across all edge definitions. The exact SVM classification results for each realization of graph construction method are depicted in Figure S2 (see Supplemental Material).

**Figure 10:**
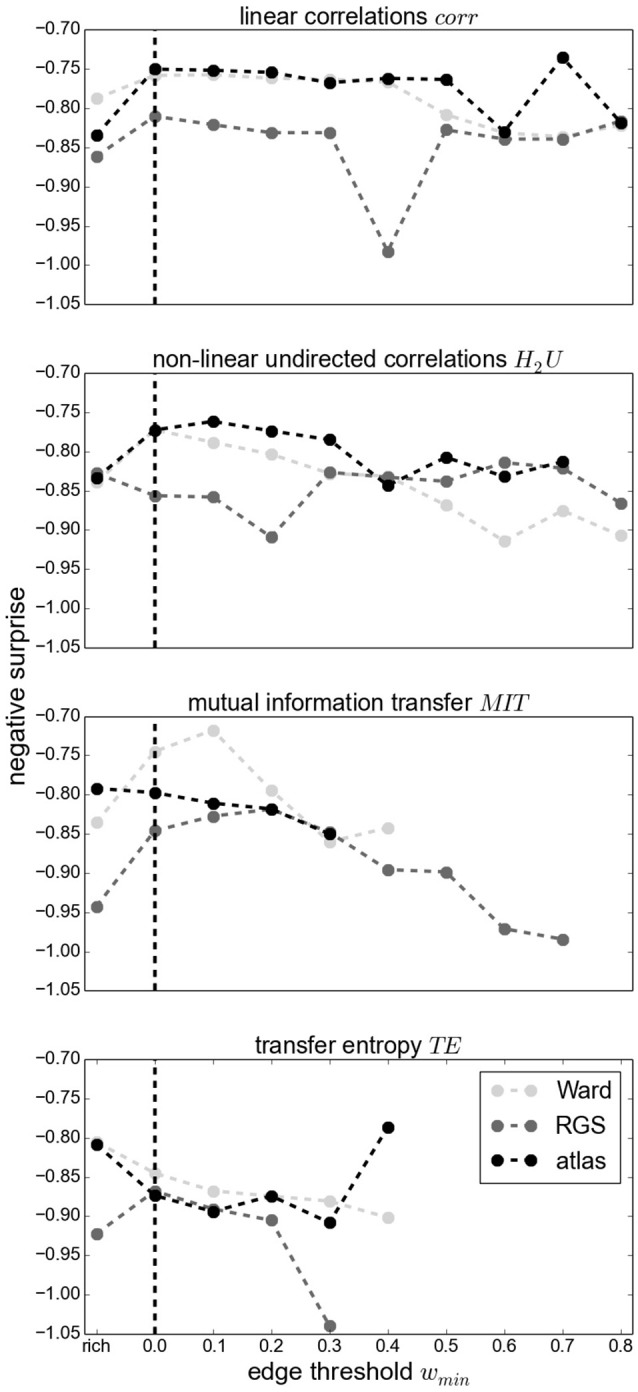
Negative surprise for different graph edge thresholds *w*min (*w*min = 0 for complete graphs, indicated by a vertical dashed line) and rich club graphs (rich) for different edge definitions: *corr* **(First Panel)**, *H*_2_*U* **(Second Panel)**, *MIT* **(Third Panel)**, *TE* **(Fourth Panel)** and different clustering methods Ward (light gray), RGS (dark gray) and atlas (black). Each dot is the result of averaging across all possible parameters of a general graph construction method (for *w*min = 0 the average across all points of a swarm in Figure 8). Since some methods yield small edge weights, some graphs become unconnected for large *w*min such that the statistical analysis is not conducted; no values are depicted in this case. Markers are connected for better visual comprehension.

Figure 10 demonstrates that thresholding graphs has only a minor effect on the negative surprise for small thresholds up to 0.2. No systematic relationship can be observed for the effect of larger thresholds; for example, increasing the threshold to 0.4 causes a decrease in negative surprise for RGS clustering with linear correlations or mutual information, but an increase for atlas clustering with transfer entropy edge detection. Likewise, the creation of highly connected and rich club sub-graphs typically decreases the negative surprise, but in some cases increases it (e.g., RGS *H*_2_*U*). Overall the highest negative surprise (−0.66) is obtained for ward4 clustering combined with *BMITU*1 thresholded at *w*min = 0.1.

These results suggest that the best combination of graph construction techniques to use for this data set is the atlas-based or ward4 clustering combined with linear correlation methods or mutual information transfer. Thresholding the graph edges, which might reduce experimental noise and does lower computational complexity, has only a minor effect on the predictive power, as long as threshold values are small. Reducing the graphs complexity via larger thresholds or extracting the rich-club of the graph should be done with care, since the results can change in either direction. Although transfer entropy yields lower negative surprises then the model-free functional connectivity measures, we would not conclude that this edge definition performs worse in general, since it achieves high values in SVM classification. It is very likely that our choice of statistical model is not ideal, and a more tailored choice would improve performance.

## 3. Discussion

In this article we have compared different techniques for constructing and analyzing graphs. By applying a statistical model, we have demonstrated a principled method for choosing a combination of techniques for a given data set. By examining the varied outcomes of the techniques, we have shown how sensitive the results of graph theoretical analyses, such as significant differences in mean properties, can be to the choice of clustering or edge definition technique.

With regards to the predictive power of the graph construction techniques, measured in terms of negative surprise, we find that Ward and atlas clustering yield the highest performance of the clustering techniques, and region growing and selection clustering (RGS) the lowest. In particular, the variant of Ward clustering that produces large clusters and small numbers of nodes (ward4) achieved the highest performance values. Analogously for the edge detection methods, we find better performance for the model-free methods (linear and non-linear correlations, mutual information transfer) than for the model-based method of transfer entropy. For this particular data set, a combination of ward4 clustering with mutual information derived edges achieves best results. Therefore, we would recommend this combination as the primary target for a more narrowly focussed investigation based on a larger data set.

The performances we obtain are above chance level but still far away from optimal prediction of the three health conditions. One reason for this sub-optimal prediction might lie in our choice of statistical model and its parameters. With our small data set (26 controls, 16 MCI, 14 AD) the model and its parameters have a high influence on the final probabilities, and thus on the performance (Porta Mana et al., 2018). We avoided tailoring the statistical model for the theoretic and practical reasons explained in section 2.3. Even if the model is not tailored, the results are consistent with the classification performance of support vector machines (see Figure 9 and Figure S2), for the model-free edge definition techniques.

It remains unclear why Ward and atlas clustering are more successful than RGS, especially in combination with model-free edge definition. One possibility is that this is related to the large variability in graph sizes generated by RGS (Figure 2). In addition, the variance of weight distributions across subjects, and the variance of the cluster distances, are much larger in RGS then in Ward clustering (Figure 3). This could be related to the variance in the number of nodes; however, choosing graphs similar in size causes even higher variances (section 2.1). Therefore we assume that the number and connectivity of the small functional units extracted by RGS are highly variable across subjects. This variance might be even higher across subjects within a health condition than across health conditions, such that changes due to AD cannot be detected. This assumption might at first glance seem to contradict the high number of significant comparisons observed (Figure 5). However, we only calculate the significance level for the means of the distributions and not their entire shape. In addition, it is likely that some graph properties correlate with the graph size, and thus that apparent significant differences in graph properties are simply reflecting significant differences in numbers of nodes detected, and do not provide further information useful for classification or understanding the nature of the disease. Further investigation is needed on this matter.

The low negative surprise of transfer entropy (*TE*) compared with other model-free functional connectivity measures might have several reasons. The comparison of the negative surprise with the support vector machine classification suggests that a better choice of a statistical model is possible: the classification results for *TE* are similar to those of the model-free measures. In *TE* the data of a certain time interval in the past is used in order to calculate how much the uncertainty of the future is reduced. Here we use the data of the last 15 s. This time period might be poorly chosen, influencing the overall negative surprise. In addition *TE* is more sensitive to short recording periods than other methods, which may well also result in a reduced performance (Pereda et al., 2005).

With regards to the robustness of the graph theoretical outcomes, we discovered that relationships between mean graph properties, such as closeness centrality, edge weight or clustering coefficient (Figures 4–6) were sensitive to choice of clustering and edge definition techniques, to parameter choices for a given technique, and to the manner in which sub-graphs were defined (thresholding value and rich club). For most relationships between graph properties *X*, we could find significant (*p* < 0.05) differences in both directions, i.e., both *X*_AD_>*X*_C_ and *X*_AD_<*X*_C_, for specific choices of clustering and edge definition technique. This strongly suggests that a degree of suspicion should be applied to studies reporting such significant differences, especially if these results are argued to give insight into how a disease affects brain properties, unless the significance level is much more compelling or the reported differences can be validated with alternate methods.

We also investigated the sensitivity to method choice of the ordering of subjects according to a graph theoretic metric (Figure 7). In this analysis, transfer entropy was the most consistent. Nevertheless, the distributions of the negative surprises is as broad for transfer entropy as for other edge definitions (Figure 8). In general, the exact parameter selection within an edge definition method causes only slight changes in the negative surprise, more crucial is the exact realization of the clustering method: ward4 clustering generally achieves a better performance then ward3 clustering. These two variants differ only in the number of predefined clusters (see Supplemental Material Figure S1). Applying a lower threshold *w*min on the graph's edge weights has little effect on the negative surprise for all methods, as long as only small weights (up to 0.2) are set to zero. Thresholding higher weights or extracting the graph's rich club has unpredictable effects on the results, and so should be used with caution (Figure 10). Atlas clustering was least consistent in the subject ordering analysis, suggesting that although it may provide a good basis for a diagnostic tool, care should be taken in reporting discoveries of particular relationships in graph properties between health conditions, as these may well turn out to be critically dependent on the edge definition method used.

Due to the intense computational requirements of the survey performed in this article, we recognize that it would be advantageous to develop heuristics for choosing between graph construction methods without performing the full calculation for each combination. Our results suggest that properties visible at the clustering stage, such as average heterogeneity, may give some indication of predictive performance: graph constructions that result in different degrees of heterogeneity between the health conditions seem to be more discriminable by the later steps of the calculation. More research is needed in this area, which is outside the scope of the current study. In addition, it is tempting to consider *t*-test results of the mean graph properties as a heuristic. Our results suggest that this approach is largely inadequate. It holds for edge definition via transfer entropy, which gives very few significant results and the negative surprise is rather small compared with the model-free edge definitions. Conversely, region growing clustering yields most significant differences but a generally poor negative surprise. This may be due to graph properties being highly correlated, and so not providing additional information to the statistical model. In addition we used the first four moments (wherever possible) in our statistical model, rather than just the mean, which may also partially account for this apparent contradiction.

In addition to considering the predictive power and robustness of graph construction techniques, we can also evaluate them according to their practicality, i.e., speed of calculation and the extent to which they are easily available in established medical infrastructure and diagnostics. In general, applying graph theoretic measures to fMRI data for improving AD diagnosis makes sense, since MRI scans are already implemented in AD diagnostics for detecting structural changes such as hippocampal dystrophy caused by AD or AD-unrelated pathology (e.g., brain tumors). Softwares such as SPM (Tzourio-Mazoyer et al., 2002) and FSL (Jenkinson et al., 2012) are frequently used in medical research and mainly support clustering that is atlas and independent component analysis based. Ward clustering, which is the fastest of all these clustering methods, is a standard hierarchical clustering method and implemented in all standard programming softwares such as Python and Matlab. The region growing algorithm is not implemented in established softwares and is also computational very demanding. Given that it does not out-perform atlas or Ward clustering, we therefore do not recommend it. For edge definition and graph properties, several software packages are available based on Matlab (Wang et al., 2014; Kruschwitz et al., 2015) or Python^1^, which provide a comprehensive range of edge definition and graph analysis methods.

In general we recommend using statistical models and not pure classifiers such as support vector machines as diagnostic tools, since statistical models calculate a probability of a diagnosis rather than assign a classification, i.e., “Given the fMRI scan, person x has a 80% probability of having Alzheimer's disease,” rather than “Given the fMRI scan, person x has Alzheimer's disease.” Probabilities can be easily combined with other probabilities of other diagnostic tests (Porta Mana et al., 2018) such as cognitive assessment, amyloid beta and tau protein occurrence in cerebrospinal fluid, blood tests, and structural MRI^2^ (Johnson et al., 2012). This allows the medical doctor to conclude, for example: “Given the results of the cognitive test *and* cerebrospinal fluid analysis *and* structural and functional MRI scan, person x has a 95% probability of having Alzheimer's disease.” After the estimation of the probability for a disease, she has to decide on a treatment, also taking into consideration such factors as “how harmful would the treatment be for a healthy person,” which can be expressed in a utility function (Porta Mana et al., 2018). In addition, the statistical model used in this work allows an estimation of how much the model can be trusted, and therefore evaluate whether the sample size is sufficiently large (Porta Mana et al., 2018).

### 3.1. Relationship to previous studies

Studies focusing on the graph properties extracted from resting-state fMRI in AD and its pre-stages generally have one of two aims. The first aim is to identify significant differences in the graph properties between health conditions, and to use these to gain insight into the effects of AD on the physical brain and its cognitive processes. These studies complement the picture revealed by investigations based on structural MRI and functional changes on the basis of EEG and MEG recordings. Typically a variety of graph properties (e.g., nodal degree, clustering coefficient, averaged shortest path, local efficiency, betweenness centrality, global efficiency, small worldness) are calculated, and used to motivate an account of how disease-related modifications to these properties result in a reduced capacity to transfer and process information.

However, such studies reveal entirely contradictory results. For example, the value of the clustering coefficient in AD with respect to controls has been reported to be increased, unchanged, and decreased, respectively (Supekar et al., 2008; Sanz-Arigita et al., 2010; Zhao et al., 2012). Analogous contradictions have been found for the comparative length of the shortest path (Supekar et al., 2008; Sanz-Arigita et al., 2010; Zhao et al., 2012). These contradictions could be caused by methodological differences or by not separating the different states of AD. Our results show ample evidence that the precise choice of graph construction techniques can easily account for contradictory findings, even for atlas based clustering, in which the number and size of clusters is held constant across all subjects (Figure 5). Evidence that the separation of different AD stages is relevant was provided by Kim et al. (2015), who demonstrated a non-monotonic behavior of global efficiency, local efficiency and betweenness centrality across different stages of AD and MCI. In our study, we could reproduce the pattern of increase and decrease of closeness centrality across conditions (Figure 4). However, we also demonstrate that the same analysis based on the rich club sub-graph yields a different pattern, and that contradictory (but significant) results can be obtained for the same graph construction techniques with different choices of threshold. We thus conclude that differences in graph properties between health conditions are currently ill-suited to provide an account of disease mechanisms in AD, unless either: (1) a specific method of graph construction can be shown to be more representative of the underlying connectivity than other methods, (2) the differences can be shown to be robust to choice of graph construction, (3) the differences can be validated by another analytical approach, or (4) the significance level is shown to be substantially more persuasive than *p* < 0.05.

The second category of studies use graph theoretical information as input for machine learning algorithms to classify the health conditions of the subjects. Note that for this purpose it is irrelevant if a difference between health conditions is not robust to method choice, as the goal is not to understand the effects of the disease but to robustly distinguish between conditions. Recent studies have reached very high performance: 100% accuracy in discriminating AD and control (Khazaee et al., 2015), and 93% for AD, MCI and control classification (Khazaee et al., 2017). In the latter work they extract more than two dozen local and global graph properties, resulting in roughly 3, 000 features, since each of the local properties is calculated for all brain areas. Only a small subset of features is then used for classification, e.g., in-degree of the left middle temporal gyrus. They found that the classification power of local graph measures is larger than that of the global ones. Local changes in graph properties that do not propagate to global mean values have also been reported for area specific (frontal cortices, parietal and occipital regions) synchronization levels (Sanz-Arigita et al., 2010).

In this work we do not compare node-specific graph properties, because Ward and RGS clustering do not result in the same spatial location of clusters across subjects. Instead, we consider, wherever possible, the first four moments of the entire distributions of graph properties. This is more information than typically used for global measures, where often only the first moment (the mean) of a graph property distribution is taken into consideration. Nevertheless, it is still possible that considering single nodes, of which some may be more damaged by AD than others, could yield a better diagnostic performance. This requires further study in a survey considering only atlas based clustering. Again, this is out of scope of the current study, but we remark that the statistical model methodology we employ here would be equally applicable to such an investigation. The advantage of taking the entire distribution lies in the possibility of using purely data driven clustering algorithms (e.g., Ward clustering) that can be substantially faster than atlas based clustering, since they do not depend on a time and memory consuming registration of the individual brain image to standard space. In addition, the global distribution is more likely to be more robust against brain morphologic abnormalities such as brain tumors or brain shrinkage, and is more stable across recording sessions (Telesford et al., 2010; Wang et al., 2014). Finally, a short recording time might be expected to have a weaker influence on entire graph property distributions then on single nodes. Thus we conclude that global measures are preferable, if a good diagnostic performance can be reached. Although the goal of this work was not classification, we note that we obtain up to (80–90%) correct classification using an off-the-shelf support vector machine on leave-one-out subsets of our data for pairwise (C-AD, C-MCI, AD-MCI) comparisons. Whether global measures can reach the impressive performance shown by Khazaee et al. (2017) can only be investigated on a sufficiently large data set, ideally with several hundred participants.

### 3.2. Limitations of this study

In each step of the graph construction and analysis pipeline (Figure 1) we set limits to the endless space of possible methods and their corresponding parameters. Here we will shortly summarize the reasons motivating the selection of the methods examined here and the exclusion of others, given the constraint of limited computational and temporal resources. As a general principle, we aimed to include the most commonly used method(s) and additional methods that we found to be reasonable, even if they are not currently frequently used.

Starting with the fMRI pre-processing, we had to decide whether to include global signal regression. The global signal (the average activity across all brain voxels) is assumed to originate partly from vascular and respiratory processes that do not represent neuronal activity. However, there is also evidence that it contains neuronal-signaling based components, since it is negatively correlated with the EEG signal and strongly correlated with the activity of the largest network in the brain (the default mode network, which plays a major role in rest state activity) when noise levels are low (Murphy and Fox, 2017). Without global signal regression, the Pearson correlation distribution derived from the signal of all voxels, or the average activity of clustered voxels, is biased to the right such that negative values are rare and small. The correction for the global signal centers this distribution, such that negative values are much more prominent. This also changes the properties of the graphs extracted from such data, for example an increase in modularity combined with fewer unconnected nodes has been reported (Schwarz and McGonigle, 2011; Hayasaka, 2013).

Speaking against global signal regression is the finding that correction for white matter, CSF and motions yield the most stable graph properties across sessions compared with additional applied global regression (Schwarz and McGonigle, 2011). In diagnostics it is important to have only small variance in the outcome across different sessions if the health condition of a subject is stable, such that small changes that indicate a worsening of the health condition can be rapidly detected. Moreover, we define the edges of our graphs as the absolute values of the functional connectivity values. As the negative part of the correlation distribution is small without global regression, different possible treatment of negative correlations (taking the absolute values or setting them to zero) should have only a small influence on the resulting graph properties, at least when the underlying functional connectivity are based on correlations. Consequently, we elect not to include global signal regression in our pipeline.

In the clustering step, the most commonly used method is to define clusters based on cortical regions defined by a brain atlas. We supplemented this with two data-driven clustering approaches: Ward clustering and RGS clustering. We selected Ward clustering, as it has been shown to perform better than alternative hierarchical clustering methods with respect to reproducibility and accuracy (Thirion et al., 2014). RGS, a method derived from image processing (Lu et al., 2003), was selected because we could adjust the method to produce functionally homogeneous clusters. In this formulation, the only free parameter of the algorithm is the minimal cluster size. For both data-driven methods, we selected parameters such that graphs did not exceed a maximal size of 1,500 nodes, due to computational limitations. We excluded clustering based on independent component analysis, because of its laborious implementation and the requirement for domain expertise to distinguish noise from activity-related components. We also excluded all clustering algorithms that do not take functional consistency into account, e.g., dividing the voxels into cuboid patches, as has been proposed for structural data (Amoroso et al., 2017).

With regards to methods for edge definition, we limit our survey to functional connectivity measures that act in the time domain and not in the frequency domain, thus omitting frequency based wavelet analysis (Supekar et al., 2008), synchronization likelihood (Sanz-Arigita et al., 2010) and coherence (Wang et al., 2014). The most commonly used and simplest functional connectivity measure is the Pearson correlation coefficient (e.g., Zhao et al., 2012), which we name *BCorrU* in our work. We also test two additional model-free and one model-based method. A further model-based method based on Granger causality was excluded because it is too computationally expensive for larger graphs (Wang et al., 2014).

A thresholding operation is often applied to graphs extracted from fMRI, setting all values below *w*min to zero. The aim of this step is to reduce experimental noise, which mainly manifests in the weaker edges, and to make the computation of graph properties computationally less demanding (Bordier et al., 2017). The threshold *w*min can be defined in several ways: it can be set arbitrarily, without satisfying a certain demand, or such that certain properties of the graphs are preserved, e.g., average number of edges per vertex (Sanz-Arigita et al., 2010), node density (Zhao et al., 2012), small world behavior (Bassett et al., 2008) or a fixed cluster coefficient. Alternatively, it can be set such that information on the network's community structure is maximized; see, e.g., Bordier et al. (2017). In a variant of the thresholding approach, it has been proposed to transform the edge weights by applying a power law (Schwarz and McGonigle, 2011). In this study, for the sake of simplicity, we examine graph properties as a function of *w*min without targeting any specific value of a graph property. Potentially, our results would reveal a different picture if *w*min was optimized for each subject to attain, for example, a specific average nodal degree. However, comparison of these two different thresholding mechanisms resulted in no major difference in the relationships of graph properties between the control and AD groups (Sanz-Arigita et al., 2010).

We do not binarize our graphs (setting all values below *w*min to zero and those above it to one) as is frequently done (e.g., Zhao et al., 2012), as this leads to a loss of information, and moreover some distributions of graph properties would become discrete (e.g., only ones and zeros for edge weights distributions), such that higher moments would be uninformative. The disadvantage of using weighted graphs lies in the limitation of possible graph properties. Most graph properties are well-defined for binary graphs and have been partly extended to weighted graphs. Here, we calculate the (normalized) weighted degree, shortest path, closeness centrality, clustering coefficient, and the modularity. We only investigate the most commonly used metrics and do not include more complex methods such as the minimal spanning tree (Çiftçi, 2011).

In addition to the restrictions of scope with regards to the examined techniques, a clear limitation of this study is the small data set. As our aim here is primarily to provide a methodology for evaluating and comparing analysis methods, rather than to draw conclusions on the effect of Alzheimer's disease on the graph properties of the cortex, a small data set is less problematic. Indeed, for the explorative survey carried out here, a large data set would have been prohibitively expensive with respect to computational resources. Moreover, many studies applying graph analysis to fMRI data are based on similarly sized data sets, which highlights the importance of raising awareness about the methodological artifacts we have identified.

The results of our survey indicate which combinations of methods are promising in view of Alzheimer diagnosis and should be investigated further in future studies based on larger data sets. Naturally, such studies could yield some quantitatively different results to those reported here, particularly with regard to the classification performance. Nonetheless, we would like to summarize some conclusions of the work that are unlikely to change with a larger data set. First, our results show that different combinations of methods can lead to contradictory findings with regard to significant differences in mean properties (section 2.2). This effect is unlikely to be resolved by a larger sample size. Second, methods showing good robustness with respect to parameter choice for a small sample size (e.g., *TE* edge definition, see Figure 7), are likely to remain robust with increasing sample size. Likewise, there is no reason to assume that methods performing well in all circumstances for the small data set, e.g., Ward clustering combined with *corr* edge definition (section 2.3), would perform worse for larger data sets. Finally, we assert that thresholding the graphs of a large data set with a small *w*min (as shown in section 2.3) would similarly not result in a sudden jump in negative surprise.

### 3.3. Application of approach to other analysis techniques

We have demonstrated a systematic, quantitative approach for comparing and evaluating sequences of algorithms that result in classification of fMRI data based on the first four moments of simple graph theoretic metrics defined on the whole graph. However, the approach we present is equally well suited for assessing pipelines based on other metrics, as we briefly outline in the following.

One possibility is to consider the graph properties of individual nodes, as these have been shown to be very informative (Xia et al., 2014; Khazaee et al., 2015; Wang et al., 2016; Dillen et al., 2017).

This entails the use of atlas based clustering. We speculate that a global analysis of graph properties would be both faster and more robust to brain abnormalities and short recording times, and so would be the preferable approach if equivalent performance levels can be attained.

A second possibility is to extend our approach to a hierarchical analysis. This could potentially be of great use, as previous studies based on PET imaging have suggested that in Alzheimer's disease, long range connections become weaker but local clustering increases (Pagani et al., 2016, 2017). These alterations would not be observable using the graph analyses so far considered, although we have taken the first step by calculating the modularity, which compares the ideal dissection of the given graph into modules with that of a random graph with similar edge weights.

To capture the graph meta-structures it is necessary to cluster graph nodes into modules, or sub-graphs. Modules can be defined either purely functionally, such that each node (ideally) has the strongest connections to the nodes in its own cluster, and the weakest connections to nodes of other clusters, or based on anatomic structures, such that nodes in a cluster are part of large, anatomo-functionally similar brain areas. Analogous to the variety of methods for spatial clustering and edge definition investigated in this study, there are many techniques used to cluster nodes into modules (e.g., k-clustering, hierarchical clustering and spectral clustering, for a review see Schaeffer, 2007 or anatomo-functional clustering, see Pagani et al., 2016), and likewise multiple options for analysing the characteristics of the resulting modular structure (e.g., module degree or participation coefficient; see Guimerá and Nunes Amaral, 2005). Such a comprehensive study is outside the scope of the current work, but could well provide great insight into health condition related alterations in the global network structure of the brain.

## 4. Conclusions

In order to achieve a robust and successful Alzheimer's disease diagnosis based on graphs extracted from fMRI data, we recommend clustering that results in rather small graphs with large clusters. Ward clustering, in which the number of clusters can be predefined, is fast, but requires programming knowledge to implement it. Atlas clustering is well established standard fMRI analysis software applications, but it is slow and might be affected by morphologic abnormalities in the brain, such as atrophy which is a common symptom of AD.

Edge weights should be calculated based on correlations or mutually information transfer, especially if a focus of the study is uncovering significant differences in mean graph properties between health conditions. We emphasize that the existence, magnitude *and direction* of such significant differences can be very sensitive to the methods chosen, and the parameterization of those methods, and so such findings should be reported with care, especially if a biological interpretation of said findings is claimed. Transfer entropy rarely gives significant results, but is more robust toward parameter changes in the algorithm and different clustering algorithms. Finding appropriate statistical models may be an additional challenge for this method.

Weak thresholding may be used for complexity reduction as it has little effect on performance. Applying a higher threshold or extracting the rich club sub-graph (The 10% of nodes with highest degree) causes unsystematic changes in the negative surprise and should therefore be used with caution, and validated against the full graph.

In summary, our quantitative evaluation and comparison of graph construction and analysis methods provides insight into how contradicting results come about in studies of graph properties of fMRI data, and identifies a number of potential methodological artifacts. Moreover, it provides a blueprint for establishing appropriate analysis pipelines, and serves as a well-founded starting point for future research on larger data sets.

## 5. Methods

### 5.1. Data acquisition

The recruitment and neuropsychological assessment of the study participants is given in Dillen et al. (2017). Demographic information is given in Table 2.

**Table 2:** Demographic information of participants.

	**Controls**	**MCI**	**AD**
Number	26	16	14
Age	62.38 [50, 73]	70 [55, 78]	71 [61, 78]
Sex	10 f, 16 m	7 f, 9 m	7 f, 7 m
Years of education	15.3 [8, 25]	12.75 [8, 21]	12.83 [7, 18]

*Average and minimal and maximal values [min, max] are given for age and years of education; female (f), male (m)*.

Anatomical MRI and resting state fMRI (rfMRI) images were obtained from a 3T MR-Brain-PET scanner (Siemens, Erlangen, Germany) in the Memory Clinic Cologne Juelich. The parameters for the single-shot echo planar imaging sequence of the functional (T2* weighted) image are the following: TR = 3, 000ms, TE = 30ms, FA = 90°, FOV = 200 × 200mm^2^, matrix = 80 × 80, voxel resolution = 2.5 × 2.5 × 2.8, 50 oblique slices parallel to the infra-supratentorial line, gap = 0.28mm, interleaved, scan time = 7min. Parameters of the high-resolution T1-weighted structural image based on a magnetization-prepared rapid gradient echo sequence: TR = 2, 250ms, TE = 3.03ms, FA = 9°, FOV = 256 × 256mm^2^, matrix = 256 × 256, voxel resolution = 1mm isotropic, 176 sagittal slices, no gap, interleaved, scan time = 314s. For more detail see Dillen et al. (2017).

### 5.2. Preprocessing of fMRI-data and extraction of cortical data

Image preprocessing is accomplished using FMRIB's Software Library tools (FSL; Woolrich et al., 2009; Jenkinson et al., 2012). We carry out the following steps for the structural T1-weighted image: skull-stripping (Smith, 2002) with bias field correction (Keihaninejad et al., 2010; Leung et al., 2011; Popescu et al., 2012) and for the functional T2-weighted image: discarding the first 10 volumes (out of 140 each taken after 3sec), motion correction (Beckmann and Smith, 2004), spatial smoothing using a 4 mm full width at half maximum Gaussian kernel, high-pass temporal filtering at 0.02 Hz and a six-parameter, rigid-body linear transformation procedure in MCFLIRT (Jenkinson et al., 2002). More details can be found in Dillen et al. (2017), where the same preprocessing is applied. In addition we carry out white matter and cerebrospinal fluid regression (FSL regfilt, MELODIC) to the functional image in order to reduce noise.

In order to extract only cortical voxels from the entire brain fMRI image, as needed for the data-driven clustering described in the next section, we first register cortical regions (frontal-, occipital-, temporal-, and insular-cortex) defined in the MNI structural atlas (Collins et al., 1995) to the structural and then to the functional space. For this registration we apply the transformation matrix obtained from registering the entire standard brain first to the individual structural brain (linear registration with FSL/FLIRT; Jenkinson and Smith, 2001; Jenkinson et al., 2002) and then to the functional space (non-linear registration with Advanced Normalization Tools, ANTs; Avants et al., 2011). In order to extract only gray matter tissue, we apply the gray matter image of the structural space (segmentation with FSL-FAST; Zhang et al., 2001) registered to functional space as described above, as a mask to the to the functional image.

### 5.3. Data-driven and atlas based clustering of cortical voxels

In order to construct graphs we cluster cortical voxels into regions using three different methods. Two of these methods, the Ward clustering and the region growing and selection algorithm (RGS) are data driven, such that only neighboring voxels with similar activity are combined into a single region. For these algorithms the number of regions per brain and the participating voxels in a region can differ for each individual and strongly depend on predefined algorithm-specific parameters. The atlas-based cluster algorithm, in contrast, produces the same number of clusters and a constant number of voxels per region across individuals, because the individual brains are mapped onto a standard brain.

#### 5.3.1. Atlas-based clustering

For each subject we linearly register the rfMRI image first to the structural, skull-removed image (image segmentation for skull removing with SPM8, Wellcome Department of Cognitive Neurology, London, UKFSL; linear registration with FSL/FLIRT; Jenkinson and Smith, 2001; Jenkinson et al., 2002) and then, through a non-linear mapping, to the MNI standard brain [non-linear registration with Advanced Normalization Tools (ANTs; Avants et al., 2011); MNI 152 standard brain, non-linear 6th generation (Grabner et al., 2006)]. Regions of interest (ROIs) of the resulting functional image in standard space are extracted such that they match the 94 regions identified by the Oxford lateral cortical atlas (regions have a probability above 50%) (Desikan et al., 2006). A demonstration of how the brain is clustered according to the brain areas is given in the first panel of Figure 12.

#### 5.3.2. Ward clustering

Ward clustering (Python: *sklearn.cluster.AgglomerativeClustering*, Pedregosa et al., 2011) is a data-driven clustering algorithm, which is initiated by defining each voxel as a cluster and then, in each iteration step, merging the two neighboring clusters (even of different sizes) that after merging show minimal intra-cluster variance compared with all other possible variations of combining two adjacent clusters. In this way, the number of clusters is reduced by one in each iteration step. In our case the clustering stops after *k* clusters (Table 3) are formed. Afterwards, we discard away all clusters that contain less then *p* voxels (Table 3). An example of the outcome of Ward clustering algorithm is depicted in the second panel of Figure 12.

**Table 3:** Parameters used for the different clustering algorithms.

	**Minimal number of**	**Number of**	**Threshold *T* of Pearson**
**Method**	**voxels per cluster *p***	**clusters *k***	**correlation coefficient**
ward1	10	5,000	–
ward2	25	5,000	–
ward3	10	2,000	–
ward4	25	2,000	–
RGS1	55	–	0.75
RGS2	50	–	0.75
atlas	–	–	–

*Ward clustering (ward), region growing and selection (RGS), atlas-based clustering (atlas)*.

#### 5.3.3. Region growing and selection

**Figure 11:**
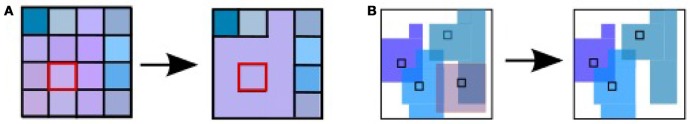
Region growing and selection algorithm. **(A)** Region growing, left: each voxel (colored squares) serves as center for a cluster, right: example of a growing region (purple), only adjacent voxels that fulfill the fusion criteria are added to the growing cluster. **(B)** Region selection. Small regions (pink) with centers overlapping with larger regions (green) get deleted (from left to right) in a iterative manner. Remaining regions can still overlap as long as their centers do not cover other regions. This illustration is in 2D for simplicity, the algorithm used for fMRI data acts in 3D following the same rules.

The region growing and selection algorithm is a modified version of the algorithm described in Lu et al. (2003). Region growing implies that each voxel serves as an initial seed (center) and neighboring voxels are added iteratively if they fulfill a certain growing criteria. (Figure 11A) The condition proposed for adding a voxel to a region is based on the Pearson correlation coefficient *R* between the averaged time-varying signals of the pre-merged region and the signal of the voxel to be tested (Lu et al., 2003). If this correlation is higher then a pre-defined threshold *T* (Table 3), the voxel is merged to the region. We tighten the growth criteria by imposing a second condition that allows the merging of voxels only if, in addition to exceeding the correlation threshold, the resulting cluster is also functionally homogeneous. Here, functional homogeneity means that the time-varying signals of all voxels can be expressed as instances of a single signal with varying levels of noise. The number of independent signals in a cluster can be estimated by the spatial functional heterogeneity *h* (Marrelec and Fransson, 2011):

(1) $$\begin{array}{r} h=n_{0}+\frac{e_{n_{0}}-b_{n_{0}}}{\left(e_{n_{0}}-e_{n_{0}+1}\right)-\left(b_{n_{0}}-b_{n_{0}+1}\right)}, \end{array}$$

where *e*_*n*_ are the eigenvalues of the *N***x***N* covariance matrix of all *N* time varing signals in a cluster that exceed the eigenvalues generated by the broken-stick model *b*_*n*_, such that $e_{n}>b_{n}=\sum_{i=n}^{N}1/i$. The index *n*_0_ accounts for the smallest eigenvalues that fulfill this inequality equation, such that *e*_*n*_0__>*b*_*n*_0__ and *e*_*n*_0+1__<*b*_*n*_0+1__. A value of *h* = 1 indicates a homogeneous cluster.

**Figure 12:**
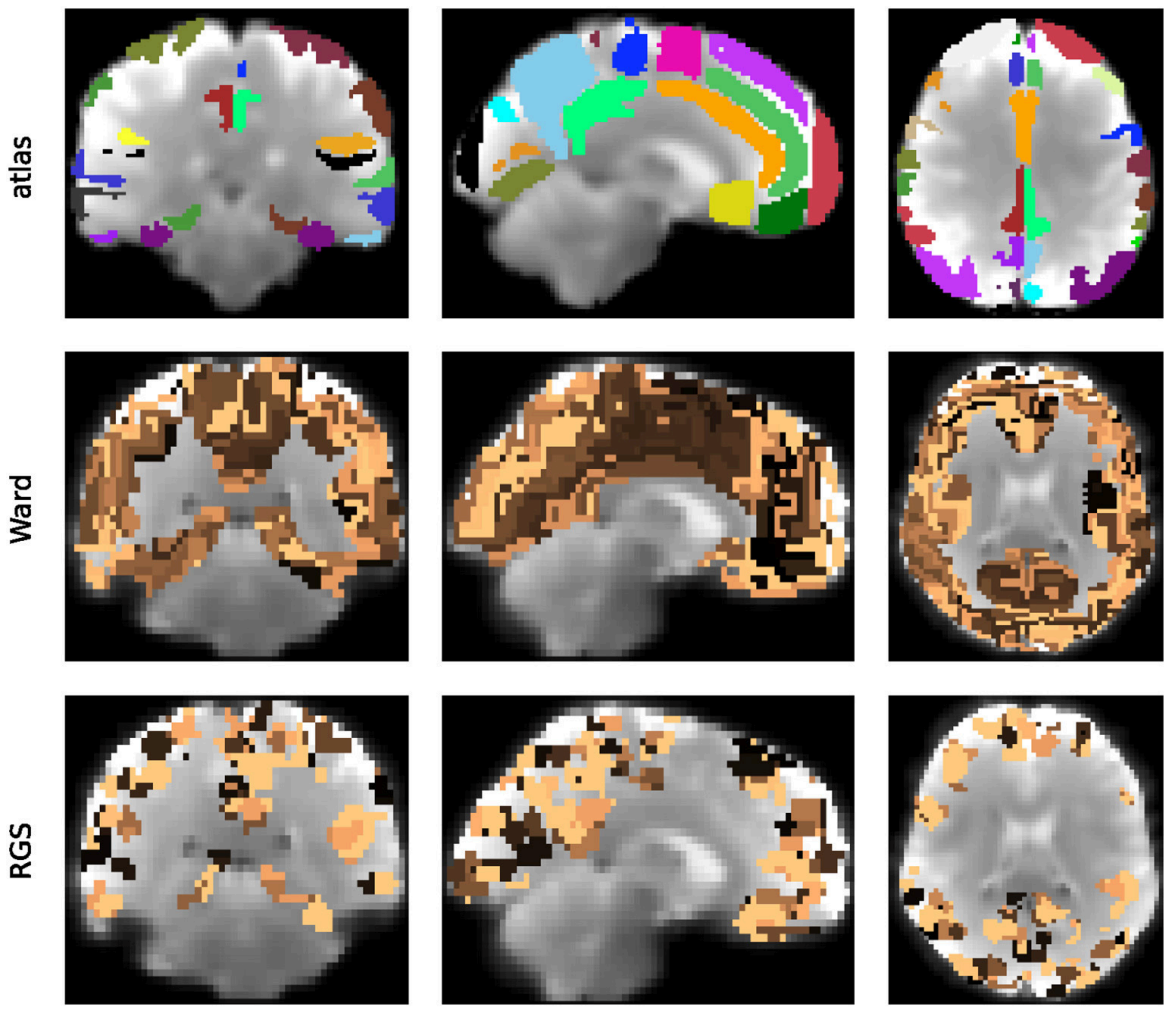
Clustering of the cortical functional image. Illustrated are the clustering outcome of the atlas **(Upper Panel)** and the Ward clustering (ward4, **Middle Panel**) and RGS (RGS1, **Lower Panel**) algorithms for frontal, sagital and horizontal brain sections (from left to right) of a randomly chosen healthy individual. Individual clusters are depicted by a randomly chosen individual color, for clustering parameters see Table 3.

The region selection algorithm iteratively selects the largest region and deletes all clusters that have their centers in that region, excluding the possibility that centers overlap with other regions. However, clusters can still overlap (Figure 11B). Applying this framework does not guarantee that clusters remain spatially connected after deleting regions with overlapping centers. Nevertheless, a check for spatial consistency reveals that only a negligible fraction of the clusters are disrupted in that way. Finally, we took only the clusters that comprised a minimum number of voxels *p* (Table 3). The outcome of RGS is illustrated in the last panel of Figure 12.

### 5.4. Edge definition

A graph consists of nodes (vertices) that are connected through edges, that might be weighted or binary and directed or undirected. We construct individual brain graphs by defining nodes that represent clusters as described in section 5.3, such that the mean activity of a cluster becomes a node attribute. We presume that all graphs are fully connected and edge weights are defined in terms of functional connectivity. Since functional connectivity can be calculated in several ways, we apply a range of different connectivity measures. In Wang et al. (2014) many such methods are evaluated, taking the structural connectivity of a toy model as reference. As a starting point, for each proposed category of functional connectivity, measured in time, we select the analysis measurement that captures structural connectivity best. We follow this strategy for all proposed measurement categories in Wang et al. (2014), leaving out only Granger causality measures, due to limited computational resources. We thus use linear and non-linear correlation (*corr* and *H*_2_) and mutual information transfer (*MIT*) for the model-free category and transfer entropy (*TE*) for the model-based category. In all groups the bivariate methods perform better then the partial ones. In conclusion we select for each of the families the bivariate implementation that can be both directed and undirected. For consistency we use the same abbreviations for the different methods as in Wang et al. (2014) and the same Matlab toolbox Mulan^3^ which they made public. Here we provide only a short description of the applied methods and more details can be inferred from Wang et al. (2014).

Linear correlation (*corr*) are measured based on the Pearson correlation coefficient (Rodgers and Nicewander, 1988) in a pair-wise manner. For directed connectivity (*BCorrD*) delays of up to 5 time steps (Table 4) are considered and the largest connectivity value is selected. We do not take into account time lags for undirected correlation (*BCorrU*).

**Table 4:** Parameters of the different functional connectivity measures.

**Method**	**Window size**	**Window overlap**	**Number of bins**	**Max. delay**
*BcorrU*1	130	–	–	–
*BcorrU*2	50	0.2	–	–
*BcorrD*1	130	–	–	5
*BcorrD*2	50	0.2	–	5
*BH*2*U*1	130	–	10	–
*BH*2*U*2	50	0.2	10	–
*BH*2*D*1	130	–	10	5
*BH*2*D*2	50	0.2	10	5
*BMITU*1	130	–	5	–
*BMITU*2	50	0.2	5	–
*BMITD1*	130	–	5	5
*BMITD*2	50	0.2	5	5
*BTEU*1	130	–	–	5
*BTEU*2	50	0.2	–	5
*BTED*1	130	–	–	5
*BTED*2	50	0.2	–	5

*Bivariate (B), undirected (U), directed (D), linear correlation (corr), non-linear correlation (H_2_U), mutual information entropy (MIT), transfer entropy (TE)*.

Non-linear correlations (*H*_2_) are based on piece-wise linear correlations of two time signals on which the non-linear curve is fitted (da Silva et al., 1989). Bivariate directed (*BH*_2_*D*) and bivariate undirected (*BH*_2_*U*) are defined as above for linear correlations.

Mutual information indicates how much information is shared between two time varying signals by means of Shannon entropy (Grassberger et al., 1991). For *BMITD1* individual histograms of two time series are contrasted to the joint histogram across different time delays. No delays are taken into account in *BMITU*.

Transfer entropy (Schreiber, 2000) describes how far in the past the activity of a node can reduce the uncertainty of the future activity of another node for which the past activity is also considered. Bivariate directed (*BTED*, Chicharro, 2011) and bivariate undirected (*BTEU*) are defined as above for linear correlations.

All methods were tested for a window size that comprises the whole time range (130 time points/6.5 min) and for a sliding window of 50 time points (2.5 min) with an overlap of 10 time points (0.5 min), see Table 4. If the methods revealed negative weights, the absolute value was considered. The resulting graphs are directed or undirected weighted graphs with values between zero and one for all methods except non-linear correlations, where values can exceed one.

**Figure 13:**
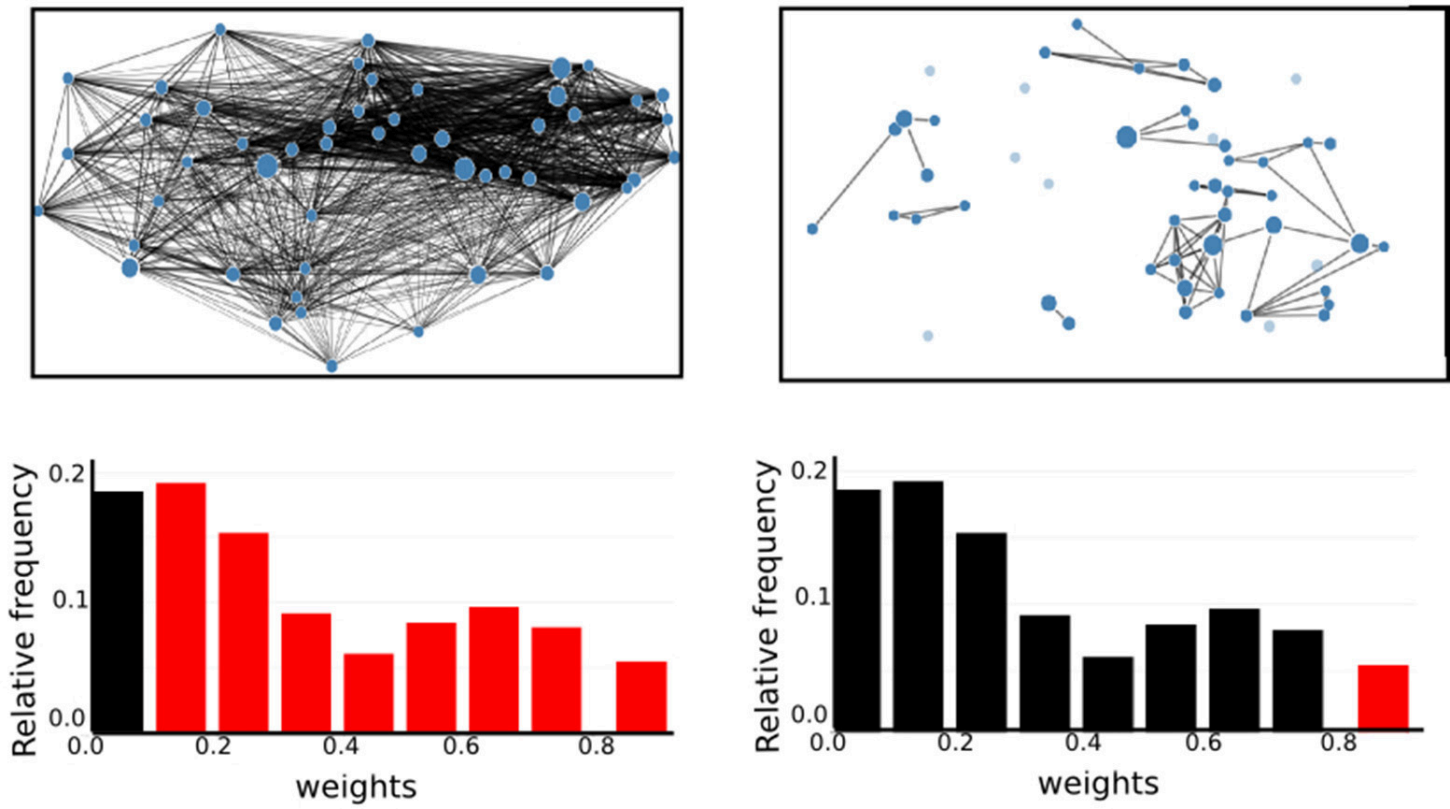
High thresholds on graph edges cause the graphs to dissociate. **(Upper Panel)**, Illustration of graph with edge weights larger then 0.1 (*w*min>0.1, **Left**) and larger then 0.9 (*w*min>0.9, **Right**). In the according weight histograms **(Lower Panel)** the red bars correspond to the edges drawn in the upper graph. Edges corresponding to the black bars are not shown.

Many studies transfer weighted graphs into binary ones by setting all values below a threshold *w*_min_ to zero and above to one e.g., Zhao et al. (2012). Following this strategy we also investigate the effect of setting all weights below *w*_min_ to zero but leaving higher weights unchanged. As far as the remaining graphs are still connected (left panels in Figure 13) and single nodes are not disconnected from the network (right panels in Figure 13) we study the disease diagnosis capacity for *w*_min_∈{0.1, 0.2, …0.7, 0.8}. In addition we extract the rich club of the graphs. The rich club is a subgraph that comprises the nodes that are most strongly connected to the network. In this work we define the rich club as the 10% of nodes with highest degree.

### 5.5. Graph properties

This section describes the different graph properties that are either characteristics of single nodes (weighted degree, closeness centrality, cluster coefficient), of pairs of nodes (shortest path) or of the entire network (modularity). In the first two cases we get a range of values for each graph. Since we do not know, which are the important features of the resulting distributions, we take the first four moments for our statistical analysis. Because graphs based on data-driven clustering contain different number of nodes and the calculated graph properties might be dependent on the number of nodes, we also include the number of nodes in the subsequent analysis (section 5.6).

#### 5.5.1. Weighted degree

The weighted degree deg_*w*_ describes how strongly a node is connected to all other vertices of the network, obeying the equation:

(2) $$\begin{array}{r} {\text{deg}}_{w}\left(v\right)=\underset{u\in V\backslash \left\{v\right\}}{\sum }w_{uv} \end{array}$$

where *w*_*uv*_ is the weight on the edge between nodes *u* and *v* of all nodes *V* in the graph. This definition implies a high dependency of the weighted degree on the number of nodes in a graph. To address this problem, we normalize the weighted degree

(3) $$\begin{array}{r} {\text{deg}}_{n}\left(v\right)=\frac{{\text{deg}}_{w}\left(v\right)}{\text{deg}\left(v\right)\cdot w_{\max}} \end{array}$$

with *w*_max_ being the maximal weight of the graph. The resulting values are between 0 and 1.

#### 5.5.2. Shortest path and closeness centrality

The shortest path dist_*w*_(*u, v*) between a pair of nodes *u* and *v* describes the path that minimizes the sum of the weights of its participating edges. A small shortest path should indicate a strong functional connectivity, therefore we consider the inverse of the graph weights for its calculation. Its computation is carried out using Dijkstra's algorithm (Rivest et al., 2000), which requires the weights to be positive.

Based on the shortest paths of a network we calculate closeness centrality *C*_*w*_(*v*) - a measure that indicates how strongly a node *v* participates in all shortest paths of the graph. It is given by:

(4) $$\begin{array}{r} C_{w}\left(v\right)=\frac{n-1}{\sum_{u\in V\backslash \left\{v\right\}}{\text{dist}}_{w}\left(u,v\right)} \end{array}$$

Here, *n* is the number of all nodes *V* in the graph.

#### 5.5.3. Clustering coefficient

The clustering coefficient *cc*(*v*) describes to what degree the neighbors of a node *v* are connected among each other and with node *v*. Since our network is weighted, we use the Zhang-Horvath clustering coefficient (Zhang and Horvath, 2005; Kalna and Higham, 2007), which is an extension to the “standard” algorithm applied to binary graphs:

(5) $$\begin{array}{c} cc(v)=\frac{{\sum^{\text{​}}}_{i\neq v}{\sum^{\text{​}}}_{j\neq i,j\neq v}{\hat{w}}_{vi}{\hat{w}}_{ij}{\hat{w}}_{jv}}{{(\sum^{\text{​}}}_{i\neq v}{\hat{w}}_{vi}){(\sum^{\text{​}}}_{i\neq v}{\hat{w}}_{vi}^{2})} \end{array}$$

for *i*, *j* neighbors of *v* and ŵ denoting the weights normalized by the highest weight in the network, such that 0 ≤ ŵ ≤ 1.

#### 5.5.4. Modularity

A graph can be partitioned into smaller components. Modularity measures the deviation of the properties of these components as compared to the components of a random graph with the same edge weights. Accordingly, the modularity of a partition *p* of a network *G* into communities *c* is given by Newman (2004):

(6) $$\begin{array}{r} Q\left(p\right)=\frac{1}{2m}\underset{i,j\in V}{\sum }(w_{ij}-\frac{{\text{deg}}_{w}(i)\cdot {\text{deg}}_{w}\left(j\right)}{2m})\delta_{c_{i}c_{j}} \end{array}$$

where δ_*c*_*i*_*c*_*j*__ is 1, if the community *c*_*i*_ of node *i* is the same as the community *c*_*j*_ of node *j*, and 0 otherwise, and $m=\frac{1}{2}\underset{i,j\in V}{\sum }w_{ij}$ is the total sum of edge weights in a network. Although there are many different definitions in literature about what a community consists of, we define a community as a group of strongly interconnected nodes that make only weak connections to other communities. In addition, a node can maximally contribute to one community. Hence we want to find the partition that maximizes modularity, which is computationally very demanding, so it is important to use a very effective algorithm. We therefore use the fast algorithm by Blondel et al. (2008), which is implemented in the Python packages community. Unfortunately this implementation is only suitable for undirected graphs, so we investigate modularity only for these type of graphs.

### 5.6. Statistical model

The generated graph data is used as input for an exchangeable parametric statistical model. Let us recall that the purpose of the fMRI scan of a patient is to give the clinician a likelihood for the patient's health condition,

(7) P(graph data from fMRI scan | health condition ^  prior info),

which she combines with the likelihoods from other tests and her initial probability assignment, to obtain via Bayes's theorem a final probability for the health condition (Sox et al., 2013):

(8) P(health condition | results of all tests ^ prior info)︷final probabilityαlikelihoods{   P(graph data from fMRI scan | health condition ^ prior info)×P(results of other tests | health condition ^ prior info)×⋯                                                      ×P(health condition | prior info).︸initial probability

The prior information also includes test results from previous patients, so that the prediction becomes more accurate and reliable, the more patients have been previously observed.

The functional dependence of the likelihood on the graph data is determined by the statistical model we use, and may be different for each health condition. The statistical model is determined by additional assumptions or hypotheses. Such hypotheses and the functional form of the likelihood may depend on the particular space of graph data (e.g., real-valued, or positive, or bounded within a finite range, or combinations thereof), and therefore on the graph construction method.

As explained in section 2.3, our purpose is to assess as far as possible the relative predictive power of the different graph construction methods. We therefore would like the functional dependence on the graph data space to be minimal. In the present study we adopt the working hypothesis that only the first and second empirical moments—means and correlations—of the graph data from past patients with the same health condition are relevant to make predictions about a new patient. This hypothesis is adopted for all graph construction methods. We also assume our initial knowledge of the graph data to be approximately invariant under rescalings of their values (Minka, 2001). Finally, we do not take into account the natural range of variability (positive, bounded, etc.) of the graph data; this choice does not seem to impact the predictive power of the model (Porta Mana et al., 2018).

These assumptions almost uniquely determine the statistical model and the likelihood (Porta Mana et al., 2018): it turns out to be a multivariate t distribution (Minka, 2001; Kotz and Nadarajah, 2004; Murphy, 2007). More precisely: select a particular health condition, e.g., Alzheimer's disease. Denote with *f*0 the *d*-dimensional vector of graph data obtained from the patient's fMRI scan via a particular graph construction method, and with (*f*_*i*_) the graph data of *n* previous patients with the selected health condition. Then the likelihood that the present patient has the selected health condition is

$$\begin{array}{l} \text{p}\left[f_{0}|\left(f_{i}\right),\kappa_{0},\delta_{0},\nu_{0},\Delta_{0},M\right]\equiv \text{p}\left(f_{0}|\kappa ,\delta ,\nu ,\Delta ,M\right)\\ \;=t\left[f_{0}|\nu -d+1,\delta ,\frac{\kappa +1}{\kappa \left(\nu -d+1\right)}\Delta \right] \end{array}$$

(9) $$\begin{array}{cc} \kappa =\kappa_{0}+n, & \nu =\nu_{0}+n, \end{array}$$

with

(10) $$\begin{array}{c} \delta =\frac{\kappa_{0}\delta_{0}+n\bar{f}}{\kappa_{0}+n},\;\Delta =\Delta_{0}+n\text{Cov}(f)\\ +\frac{\kappa_{0}n}{\kappa_{0}+n}(\bar{f}-\delta_{0}){\left(\bar{f}-\delta_{0}\right)}^{⊤}, \end{array}$$

where t is a multivariate t distribution with ν−*d*+1 degrees of freedom, mean δ, and scale matrix $\frac{\kappa +1}{\kappa \left(\nu -d+1\right)}\Delta$, and

(11) $$\begin{array}{c} \bar{f}:\;=\frac{1}{n}\sum_{i}f_{i},\;\;\text{Cov}(f):\;=\frac{1}{n} \end{array}{\sum_{i}\left(f_{i}-\bar{f})(f_{i}-\bar{f}\right)}^{⊤}$$

are the empirical mean and covariance matrix of the previous graph data.

The parameters κ0, ν0, δ0, Δ0 should reflect our initial knowledge of the graph parameters. For the reasons explained above and in section 2.3, we fix one set of values identically for all graph construction methods: κ = 1, (δ0)_*a*_ = 0.5, Δ0 = 2.5*I*, where *I* is the identity matrix. These values yield an initial distribution (before any data from previous patients) centered on positive values of unit order of magnitude, as all the graph data indeed are for each graph construction method.

### 5.7. Supportive evaluation measures of graph construction methods

#### 5.7.1. Significance test

We measure the significance level of the mean values of a graph property distribution between pairs of the three healthy conditions (control-AD, control-MCI, MCI-AD) based on the Student's *t*-test, if variances are equal (*F*-test), and Welch's *t*-test otherwise. The underlying null hypothesis is that the means of the two data arrays are assumed to be equal, which is rejected for *p*-values smaller then 0.05.

#### 5.7.2. Dendrograms of subject order

Subjects indexed from 1 to 56 (total number of participants) across all health conditions are ordered according to the mean values of a given graph property distribution. The indices of the ordering (the rank) calculated for each graph construction method is then used in order to construct the dendrogram. In the dendrogram, the Euclidean distance between two indices arrays is indicated by the height of the top of the U-link linking the two arrays. In addition, arrays with a small distance are clustered together.

#### 5.7.3. Support vector machines

For all complete graphs constructed by all different graph construction methods, we apply a support vector classification (Python: *sklearn.svm.SVC*) on each pair of health conditions (control-AD, control-MCI, MCI-AD). Hereby we choose the graph properties such that the performance of the algorithm maximizes. We use the default parameters and do not optimize performance by varying the kernel coefficient or the penalty parameter of the error term.

## Ethics statement

This study was part of a larger study, which was approved by the local ethics committee, in accordance with the declaration of Helsinki and performed after informed written consent of each participant. Healthy participants were reimbursed. AD patients were not reimbursed since imaging was part of their diagnostic procedures. We did, however, pay for and organize their traveling costs and lunch.

## Author contributions

CB constructed the graphs and calculated and analyzed the graph properties. She also applied the statistical analysis, formulated together with PP, to the data. KD, HJ, NR, BvR, JD, OO, K-JL, GF and JK contributed to the conception of the study design and recruited patients. KD, NR, BvR, and JD organized and performed fMRI scanning. KD and HJ applied primary preprocessing to the fMRI data. The manuscript was written by CB, AM, and PP, with additional editing by HJ and JK.

### Conflict of interest statement

The authors declare that the research was conducted in the absence of any commercial or financial relationships that could be construed as a potential conflict of interest.

## Footnotes

^1^https://github.com/dpisner453/PyNets.

^2^https://www.alz.org/research/diagnostic_criteria/.

^3^https://github.com/HuifangWang/MULAN.
