# Supplementary material for: On the Extraction and Analysis of Graphs From Resting-State fMRI to Support a Correct and Robust Diagnostic Tool for Alzheimer's Disease
Source: Front Neurosci. 2018 Sep 28;12:528. doi: 10.3389/fnins.2018.00528 (PMC6172342; doi:10.3389/fnins.2018.00528)
Supplement: Supplementary file 1 [file Data_Sheet_1.pdf]

Supplementary Material:
Article Title

1 SUPPLEMENTARY TABLES AND FIGURES

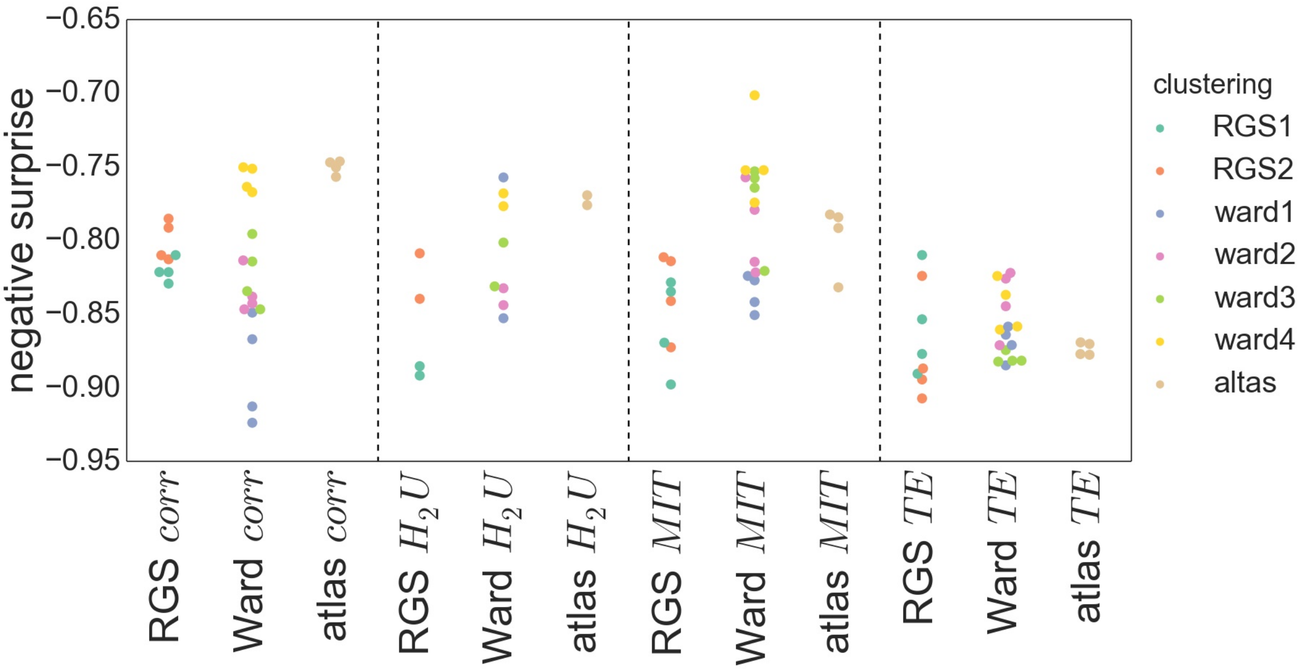

Figure S1: Negative surprise of the different graph construction methods. Figure identical with Fig.8 but with different clustering submethods depicted in different colors (see legend) .

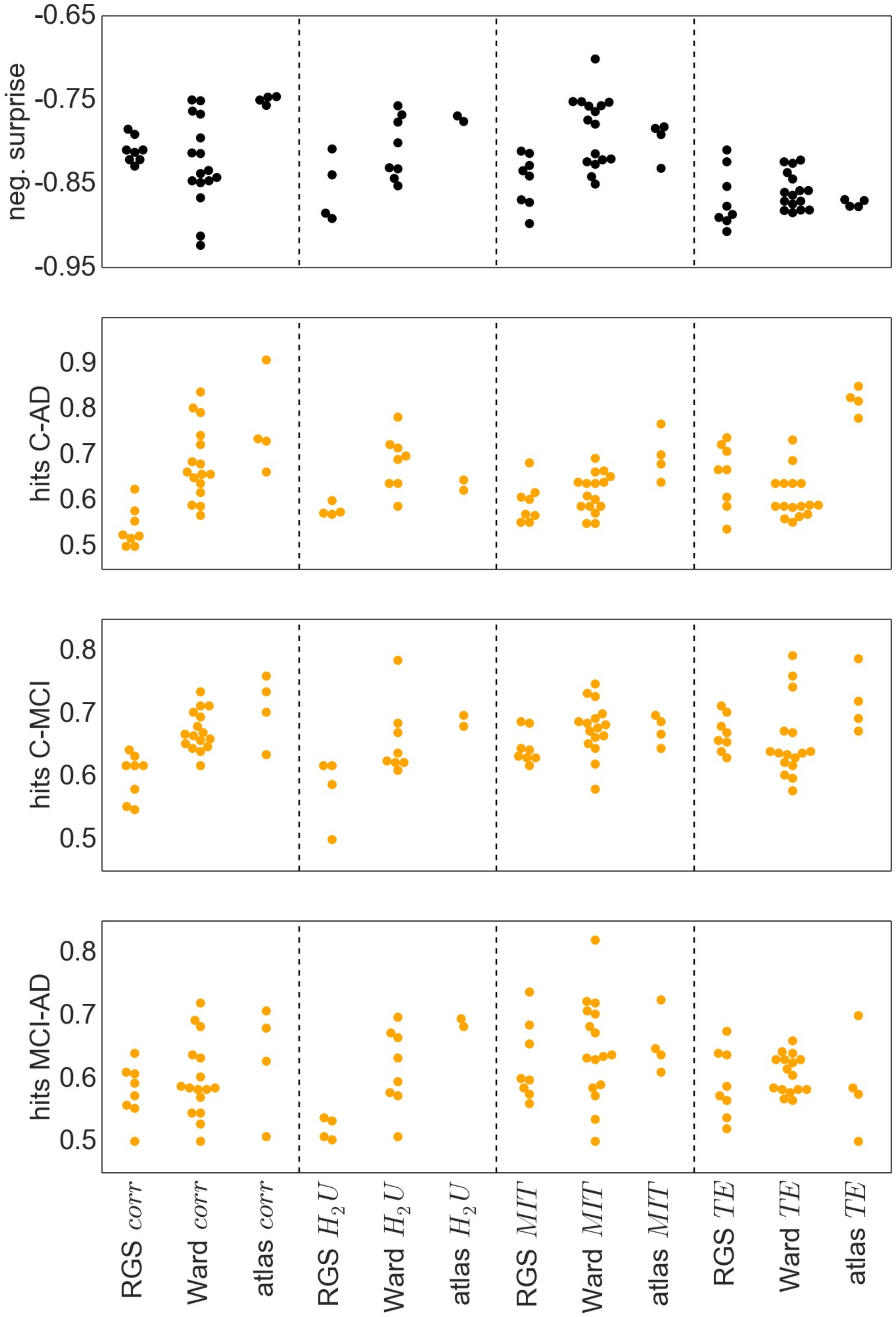

Figure S2: Negative surprise and classification results based on support vector machines. Negative surprise as depicted in Fig.8. **(First Panel)**. Correct hits based on support vector machine classification for control-AD **(Second Panel)**, control-MCI **(Third panel)** and MCI-AD **(Last panel)**.
